# Concepts and Methods to Access Novel Antibiotics from Actinomycetes

**DOI:** 10.3390/antibiotics7020044

**Published:** 2018-05-22

**Authors:** Joachim J. Hug, Chantal D. Bader, Maja Remškar, Katarina Cirnski, Rolf Müller

**Affiliations:** 1 Department Microbial Natural Products, Helmholtz-Institute for Pharmaceutical Research Saarland (HIPS), Helmholtz Centre for Infection Research (HZI) and Department of Pharmacy, Saarland University, Campus E8.1, 66123 Saarbrücken, Germany; joachim.hug@helmholtz-hzi.de (J.J.H.); chantal.bader@helmholtz-hzi.de (C.D.B.); maja.remskar@helmholtz-hzi.de (M.R.); katarina.cirnski@helmholtz-hzi.de (K.C.); 2 German Center for Infection Research (DZIF), Partner Site Hannover-Braunschweig, 38124 Braunschweig, Germany

**Keywords:** metagenomics, rare actinomycetes, dereplication, metabolomics, genome mining, natural products

## Abstract

Actinomycetes have been proven to be an excellent source of secondary metabolites for more than half a century. Exhibiting various bioactivities, they provide valuable approved drugs in clinical use. Most microorganisms are still untapped in terms of their capacity to produce secondary metabolites, since only a small fraction can be cultured in the laboratory. Thus, improving cultivation techniques to extend the range of secondary metabolite producers accessible under laboratory conditions is an important first step in prospecting underexplored sources for the isolation of novel antibiotics. Currently uncultured actinobacteria can be made available by bioprospecting extreme or simply habitats other than soil. Furthermore, bioinformatic analysis of genomes reveals most producers to harbour many more biosynthetic gene clusters than compounds identified from any single strain, which translates into a silent biosynthetic potential of the microbial world for the production of yet unknown natural products. This review covers discovery strategies and innovative methods recently employed to access the untapped reservoir of natural products. The focus is the order of actinomycetes although most approaches are similarly applicable to other microbes. Advanced cultivation methods, genomics- and metagenomics-based approaches, as well as modern metabolomics-inspired methods are highlighted to emphasise the interplay of different disciplines to improve access to novel natural products.

## 1. Introduction

Antimicrobial resistance (AMR) gains more and more public attention as one of the biggest threats to prevention and treatment of an increasing number of infections. In January 2018, the WHO released a report on global antibiotic resistance crisis. For this report, the new Global Antimicrobial Surveillance System (GLASS) implemented in May 2015 was used to support standardised AMR surveillance globally. The main objective of that study has been tracking the resistance-related issues of medicines in use for treatment of hospital and community acquired infections across the 52 participating countries. The survey focus is on *Escherichia coli*, *Klebsiella pneumoniae*, *Staphylococcus aureus*, *Streptococcus pneumoniae*, *Salmonella* spp., *Neisseria gonorrhoeae*, *Shigella* spp. and *Acinetobacter baumanii*. According to the report, *N. gonorrhoeae* is evolving into a superbug, as it is resistant to 3rd generation cephalosporins and fluoroquinolones and is classified by the WHO Priority Pathogens List of antibiotic-resistant bacteria. *Salmonella* spp., *S. aureus* and *N. gonorrhoeae* were prioritised for research and development of new and effective antibiotic treatments, while *A. baumanii*, *E. coli* and *K. pneumoniae* are classified as critical priority and *Shigella* spp. along with *S. pneumonaiae* are classified as medium priority [1]. Obviously, new lead compounds exhibiting lack of cross-resistance with known antibiotics are urgently searched for.

Between 1950 and 1960—the so-called “golden age of antibiotics” [2]—microbiology and chemistry went hand in hand; bacteria were cultivated and their secondary metabolites extracted to yield a large number of compounds with antibacterial activity. Among these bacteria the genus *Streptomyces* has provided numerous novel bioactive molecules, not just antibiotics but also antifungals, antiprotozoals and antivirals [3]. However, the discovery of novel drugs derived from streptomycete secondary metabolites has continued to steadily decrease since, reflected in a 30% drop of natural product-based drugs in clinical studies between 2001 and 2008 [4]. Natural product research was widely abandoned by pharmaceutical industry due to severe rediscovery issues; in addition, challenging isolation of new producers and low production concentrations contribute to the more difficult access to novel scaffolds with promising activity using well-known natural product sources [4]. In response to the growing needs of medicine for new antimicrobial compounds, the focus in natural products discovery shifted towards underexplored habitats, especially marine and extreme environments. Encouragingly, rare genera of actinomycetes—in general considered as non-streptomycetes—and novel *Streptomyces* isolates found in these habitats have already afforded the discovery of novel antimicrobials with unique chemical moieties, confirming that microbial natural products are still a promising source for drug discovery [5,6,7,8]. In particular, non-streptomycetes belonging to genera such as *Micromonospora*, *Nocardia*, *Actinomadura*, *Actinoplanes, Streptoverticilllium* and *Saccharopolyspora* have been found to produce chemically unique antibiotics featuring potent activities [8], such as abyssomicins [9] and proximicins [10] from *Verrucosispora* strains. Consequently, isolation of natural products from rare actinomycetes already led to the discovery of approximately 2250 new bioactive secondary metabolites until 2005 [11,12]. Bioinformatic analysis of genomes from long known actinomycetes—for instance, the first whole genome sequenced actinomycete *Streptomyces coelicolor* A3 (2) [13] known to produce only a few secondary metabolites until then—revealed that many secondary metabolite genes are most likely not expressed under conventional laboratory conditions or their corresponding products cannot be detected with widely used analytical methods. This discrepancy translates into a “silent” potential of the microbial world for the production of yet unknown natural products. However, despite these encouraging insights, the majority of nature’s microbial biosynthetic potential will remain elusive, if natural product research continues to rely exclusively on a classical culture-dependent discovery platform. Efforts must be intensified to exploit the majority of bacteria not cultured under laboratory conditions to date, and some suitable concepts and methods for that purpose have emerged recently [14].

### Outline of this Review

In light of the numerous available review articles covering microbial natural products research—including highly specialised articles diving into specific sub-topics in much detail—we here aim to present a summary of selected technical and conceptual proceedings in the field of natural product research during the last decade. We focus on examples from the world of the actinomycetes although conceptual developments using alternative producers have significant impact on this field [15,16,17,18,19]. The idea of exploring new habitats harbouring rare actinomycetes and their subsequent isolation and cultivation utilising novel cultivation methods serves as a starting point in Section 2. If large-scale cultivation is difficult to achieve or even not possible, culture-independent approaches are required to extend the number of microorganisms accessible for the discovery of novel natural products. Section 3 therefore outlines examples of natural product discovery by metagenomics and heterologous expression of selected pathways as an alternative means to investigate the uncultured environmental diversity and access its hidden natural product potential. Metagenomics mainly relies on prior examination of biosynthetic gene clusters with *in silico* bioinformatics tools. Hence, Section 4 deals with genomics-based approaches; in particular, genome mining to access silent biosynthetic pathways in cultured actinobacteria will be highlighted. Section 5 emphasises developments in analytical chemistry and the increasing use of metabolomics-inspired methods by combining data-driven and experimental approaches. The latter approaches today allow for the prioritisation of minute amounts of compounds that escaped detection previously.

## 2. Exploring New Habitats

**Figure 1:**
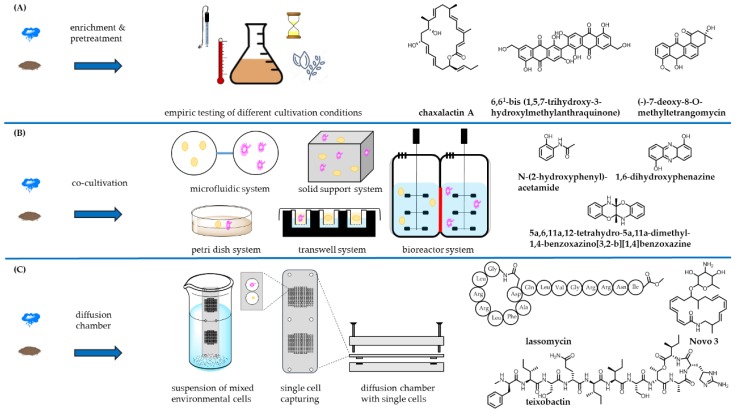
Scheme of isolation strategies, partly adapted from Nichols et al. [33] and Goers et al. [39]. (**A**) Soil sample or marine sample undergoes enrichment and/or pretreatment to increase the chance to isolate new species and/or reduce undesirable background from previously isolated strains. In general, empirical established methods for fermentation varies in incubation time, media composition, additives, pH and temperature to enable growth of desirable strains. Chaxalactin A produced by *Streptomyces* sp. C34 [26,27], 6,6^1^-bis (1,5,7-trihydroxy-3-hydroxylmethylanthraquinone) produced by *Streptomyces* spp. ERI-26 [29,30] and (-)-7-deoxy-8-*O*-methyltetrangomycin from *Nocardiopsis* sp. HR-4 [28] are examples for novel metabolites found using this conventional method. (**B**) Soil sample or marine sample is co-cultivated with other microorganisms to promote culturable isolates or to stimulate the secondary metabolism. Co-cultivation is categorised in microfluidic systems, petri dish co-culture systems, co-cultures on solid supports, co-culture systems using bioreactors and transwell systems [39]. Co-cultivation of *Actinokineospora* sp. EG49 and *Nocardiopsis* sp. RV163 induces the biosynthesis of three natural products namely *N*-(2-hydroxyphenyl)-acetamide, 1,6-dihydroxyphenazine and 5a,6,11a,12-tetrahydro-5a,11a-dimethyl[1,4]benzoxazino[3,2-b][1,4]benzoxazine [40]. (**C**) Soil sample or marine sample is used to create a suspension of mixed environmental cells. The isolation chip (iChip) plate is immersed into this suspension to capture (on average) a single cell. Covered with an upper and lower plate, the assembled iChip provides a miniature diffusion chamber for each single cell [33]. NOVO 3 [36], the cyclic peptide lassomycin [37] and the antibiotic teixobactin [38] were isolated from strains obtained by the iChip technology.

Because of over-exploitation of terrestrial streptomycetes in almost all pharmaceutical and agrochemical companies as well as numerous academic groups, in recent years, the search for new bioactive molecules has moved towards their marine relatives [20], taxa found in the extreme environments [21], endophytic species [22] and on non-*Streptomyces* actinomycetes. Especially marine ecosystems represent one of the richest and underexploited habitats with great microbial diversity showing potential for discovery of novel and chemically diverse antimicrobial compounds [23]. Progress in the beginning of this millennium in the field of selective isolation methods has been achieved, improving access to these diverse actinomycetes. Since *Streptomyces* are the most dominant genus within the actinomycete group, it is necessary to increase chances for the isolation of desirable new species (enrichment) by reducing undesirable background, i.e., previously isolated species (pretreatment) [24]. A combination of thermal pretreatment, beneficial to promote the spore germination and improve the selective isolation of rare actinomycetes and chemical techniques to eliminate bacterial contaminants has proven successful for isolation of some rare actinomycetes [24]. An advanced method of pretreatment applies polyvalent bacteriophages specifically targeting different unwanted background bacteria [25]. Traditional enrichment is based on simple trial and error methods using different chemical and physical treatments to unearth the uncultured bacterial majority. Chaxalactin A–C production—a rare class of 22-membered macrolactone polyketides produced by *Streptomyces* strain C34 which was isolated from the Chilean hyper-arid Atacama Desert soil [26,27]—was highly dependent on the culture medium used for its growth, highlighting the influence of the culture medium on growth and secondary metabolism of actinomycetes. Other recent examples for the empiric progress in isolation of actinomycetes is the halotolerant *Nocardiopsis* sp. HR-4 strain producing (-)-7-deoxy-8-*O*-methyltetrangomycin [28] and the non-extremophilic *Streptomyces* spp. ERI-26 producing 6,6^1^-bis(1,5,7-trihydroxy-3-hydroxylmethylanthraquinone) [29,30]. The reason bacteria are remaining “unculturable” is that the growth under laboratory conditions fails to mimic essential aspects of the environment, e.g., the absence of competitors [31]. One common method for simulation of environmental conditions uses diffusion chambers to enable the exchange of nutrients and growth factors within the two chambers while separating the isolates of interest from their competitors. Recovery rates up to 40% have been achieved, comparing the number of growing microcolonies by microscopic counts of cells in the initial inoculum, whereas the same inoculum on standard petri dishes yielded only a very low recovery rate of 0.05% [32]. A further developed and miniaturised *in situ* cultivation platform based on diffusion chambers is used for an isolation chip (iChip). The iChip is composed of several hundred miniature diffusion chambers, of which each is inoculated with an individual environmental cell [33]. To capture on average a single cell, the through-holes of the iChip plate are immersed into a suspension of mixed environmental cells. Covered with an upper and lower plate, the assembled iChip provides a miniature diffusion chamber for each single cell (Figure 1). This technology platform has shown the great potential for upscaling the throughput and improvement of the isolation of previously uncultured bacteria in general. The polycyclic xanthone antibiotics neocitreamicins from *Nocardia* strain G0655 [34] along with other examples described in literature, are showing that simulation of the original environment accomplished the isolation of novel strains [35]. The glycosylated macrolactams NOVO3 and NOVO4 from the strains *Streptosporangium* P1532 and *Amycolatopsis* Z0363, respectively [36], the ribosomal 16-membered cyclic peptide lassomycin from *Lentzea kentuckyensis* [37], and the antibiotic teixobactin from the new β-proteobacterial species *Eleftheria terrae* [38] have been isolated by using the iChip technology.

These case studies demonstrated how environmental conditions facilitate access to the diversity of yet uncultured microorganisms [32]. An alternative method for separation of uncultured bacteria is encapsulation of single bacterial cells in microdroplets of solidified agarose [41,42,43]. To acquire insights into metabolic requirements a metatranscriptomic approach can be utilised. High-throughput sequencing of RNA transcripts enables to access expressed genes of growing bacteria that are not culturable in large-scale. Following this metatranscriptomics-based method, an uncultured *Rikenella*-like bacterium in the leech gut was found to utilise mucin as a carbon and energy source [44].

**Figure 2:**
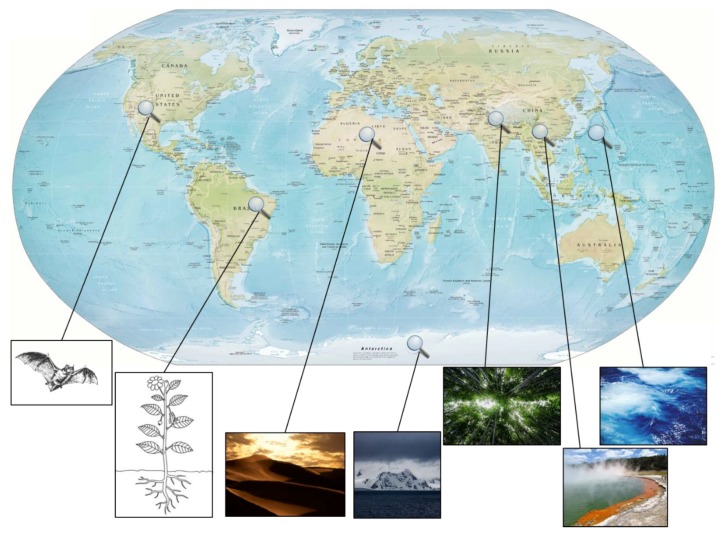
Underexploited habitats of actinobacteria attracted more attention for microbial natural product discovery. Currently, oceans [54], deserts [55], mountains [30] and Antarctica [56] ranges together with hot springs [57] and endophytes [58] and symbionts [59] are focuses of the search for new bioactive compounds.

A different approach to simulate authentic circumstances in the environment comprises co-cultivation of different species derived from the same environment, demonstrating significant influence on the production of secondary metabolites [45,46,47]. The main co-cultivation systems can be categorised by the type of technology used in microfluidic systems, petri dish co-culture systems, co-cultures on solid supports, co-culture systems using bioreactors and transwell systems for co-cultivation, allowing asymmetric levels of separation between various numbers of populations [39]. Co-cultivation of *Actinokineospora* sp. EG49 and *Nocardiopsis* sp. RV163 induces the biosynthesis of three natural products namely *N*-(2-hydroxyphenyl)-acetamide, 1,6-dihydroxyphenazine and 5a,6,11a,12-tetrahydro-5a,11a-dimethyl[1,4]benzoxazino[3,2-b][1,4]benzoxazine [40]. A hypothesised model of mechanisms on the molecular level tries to explain the reason for the necessity of co-cultivation; i.e., neighbouring bacteria are decreasing oxidative, metabolic and environmental stress, provide common nutrients or other growth factors and siderophores in order to bind and solubilise Fe^3+^ [31]. The Black Queen Hypothesis, a theory of reductive evolution, tries to explain how selection leads to helper-dependent bacterial isolates, which lost the ability to perform essential processes on their own. In conclusion the theory states that the “loss of a costly, leaky function is selectively favoured at the individual level and will proceed until the production of public goods is just sufficient to support the equilibrium community” [48]. Similar observations hold true for endosymbiotic marine bacteria, responsible for the prolific production of secondary metabolites [49]. Among the underexplored habitats, analysis of marine ecosystems has flourished (Figure 2). New actinomycetes were isolated from marine sediments, water columns at various depths and even from marine fauna. Screening of 20 marine samples from marine environments in Egypt, employing different methods for selective isolation, has yielded 112 *Streptomyces* isolates. Up to 76% of the isolates displayed activities against tested Gram-positive pathogens, up to 41% against Gram-negative pathogens, 32% against yeasts and 28% against fungi. Although depending on the type of media used, 28 isolates showed activity against methicillin-resistant *S. aureus* (MRSA), i.e., 25% of all obtained isolates. In addition, all *Streptomyces* strains showed significant nematicidal activity against second stage (J_2)_ juveniles of the root-knot nematode *Meloidogyne incognita,* making them a potential candidate for use in agriculture [50]. However, the value of these observations has to be confirmed as the molecular structures of the bioactive compounds have yet to be elucidated. The one strain–many compounds (OSMAC) approach is a strategy to overcome the limited expression of biosynthetic pathways in microbes. Prioritised strains are cultured in a variety of different media compositions and fermentation conditions to maximise the amount and diversity of produced compounds [51]. The use of OSMAC approach has enabled the recovery of a new thiopeptide antibiotic, TP-1161, from marine sediment derived *Nocardiopsis* isolate. The antimicrobial profile revealed no anti-Gram-negative activity; however, the activity against Gram-positive clinical isolates was comparable to the one of vancomycin [52]. Another example for the OSMAC approach is the isolation of the anti-MRSA and anti-vancomycin-resistant *Enterococcus* armeniaspirols produced by *Streptomyces armeniacus* DSM 19369. The armeniaspirols are only produced by the “well-characterised” *S. armeniacus* when cultivated on a malt-containing medium [53].

**Figure 3:**
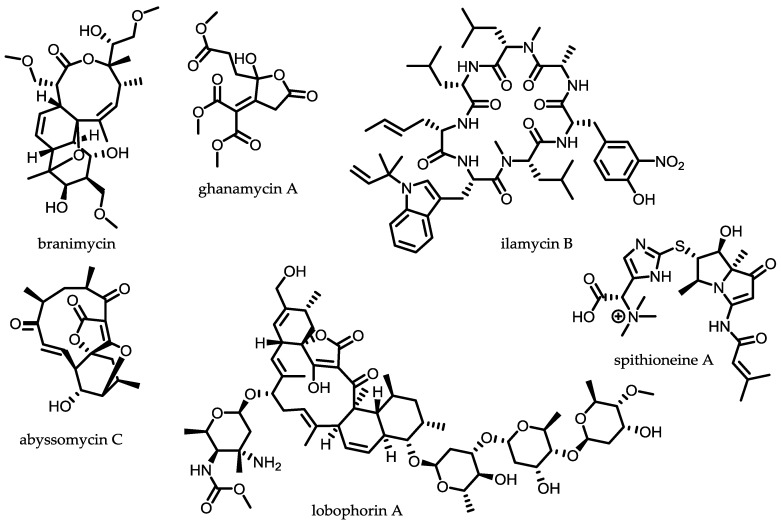
Novel natural products deriving from new habitats.

One of the more recent examples is the isolation and cultivation of the abyssal actinobacterium *Pseudonocardia carboxydivorans* M-227, isolated at 3000 m depth in water column of the Avilés submarine canyon. It produces the two new antibiotics branimycin B and C (Figure 3). Branimycins are structurally related to nargenicins and are mainly active against *S. aureus*. However, both compounds were found to be moderately active against not only *S. aureus* (MIC values 32–64 μg/mL) but also Gram-negative bacteria (MIC values 20–80 μg/mL). It is important to point out that they exhibited activity against MRSA, several clinical *S. aureus* isolates, *E. faecalis* and even *H. influenzae* ATCC 49247 [60]. Contrarily, the novel γ-butyrolactones ghanamycin A and B, isolated from a marine-derived strain *Streptomyces ghanaensis* TXC6-16, only displayed weak anti-Gram-negative activity against *Pseudomans syringae*, *Erwinia* sp. and selected plant pathogens such as *F. oxysporum*, *A. solani* and *P. oryzae* [61]. Lobophorin E and F, new spirotetronate antibiotics, exhibited moderate antimicrobial activity against *B. thuringiensis* SCSIO BT01, *S. aureus* ATCC 29213 and *E. faecalis* ATCC 29212. Interestingly lobophorin F also exhibits moderate cytotoxic activities on human tumour cell lines SF-268, MCF-7 and NCI-233 H460, emphasising the influence of chemical modifications on the observed bioactivity [62]. Spithioneine A and B are structurally unique bohemamine-type pyrrolizidine alkaloids from a marine isolate *Streptomyces spinoverrucosus*. They are especially interesting as they incorporate ergothioneine into a polyketide. Ergothioneine exhibits a wide variety of activities, such as inhibition of oxidative stress, promotion of neuronal differentiation and metal ion chelation, so it will be interesting to see the antimicrobial profile of these novel compounds incorporating this chemical moiety [63].

The question if marine derived small molecules chemically differ from those produced by terrestrial strains remains a matter of debate among natural product researchers. The famous abyssomicin was first thought to be exclusively produced by a marine streptomycete. However, it was later shown to be produced by a terrestrial streptomycete [9,64,65,66] as well. Probably the best known example that marine and terrestrial actinomycetes are producing similar lead compounds is shown by salinosporamide A from *Salinispora tropica* [67], featuring high structural similarity to the cinnabaramides A−G, isolated from the terrestrial *Streptomyces* sp. DSM 15324 [68]. Recently isolated ilamycins, produced by a *Streptomyces atratus* SCSIO ZH16, a strain isolated from South China deep-sea sediment (Figure 2) [54], carry two unusual building blocks, l-3-nitro-tyrosine and l-2-amino-4-hexenoic acid. New ilamycins produced by a genetically modified strain show very good anti-tuberculosis activity, with the MIC values against *M. tuberculosis* H37Rv in the single-digit nanomolar range (9.8 nM) [54].

### 2.1. Extreme Environments as a Rich Source for Novel Strains

Soil samples from extreme environments such as deserts and samples collected from higher altitudes offer potential to isolate many previously not cultured bacteria. Soil sample from Indian mountains uncovered a new *Streptomyces* sp. isolate, ERI-26, which produces a novel anthraquinone (Figure 2) [30]. The compound exhibits weak antibacterial activity (anti-Gram-positive and anti-Gram-negative activity >62.5 µg/mL) and moderate to good antifungal activity, with MIC values as low as 3.9 µg/mL [29]. Screening of the Egyptian desert revealed that 32 out of 75 isolated strains were active against the tested pathogenic organisms and thus showing that these extremophiles are metabolically active. According to the antimicrobial activities, 12.5% of the isolates were active against Gram-positive as well as Gram-negative bacteria and yeast strains [7]. However, without correlation of these observed activities to any known or unknown compounds, the authentic benefit of these findings is hard to evaluate. Non-*Streptomyces* actinomycete *Saccharotrix* SA198 isolated from Saharian soil has also lead to the discovery of two novel antibiotics A4 and A5 (Figure 2) [55]. Both exhibited good anti-Gram-positive and anti-Gram-negative activity (MIC values between 10 and 50 µg/mL), as well as very good antifungal profile (MIC values between 1–5 µg/mL), but no activity against yeast [55]. Furthermore, the non-*Streptomyces* actinobacterium isolate LAM143cG3 was obtained from an underexplored sebkha lake (dry lakebed consisting primarily of salt) of Kenadsa (Bechar, Southwestern Algeria). Based on the chemical and morphological characteristics, this isolate was identified as a member of the genus *Spirillospora.* Agar diffusion tests showed that crude extracts are primarily and only moderately active on Gram-positive bacteria and have only weak activity on Gram-negative bacteria [69]. Similar antibiotic bioactivity profile was exhibited by the halotolerant *Nocardiopsis* sp. HR-4 recently isolated from salt lake soil sample in Algerian Sahara [28], however the activities were not connected to known or novel natural products. One of the more promising compounds is actinomadurol, also isolated from a rare actinomycete *Actinomadura* strain KC 191 as it exhibits potent activity against *S. aureus*, *K. rhizophila*, and *P. hauseri* (MIC = 0.39–0.78 μg/mL) [70]. Actinomadurol is not inhibiting the growth of various tested cancer cell lines, even at high concentrations (IC_50_ > 100 μg/mL). Genera *Brevibacterium*, *Gordonia*, *Micromonospora*, *Arthrobacter*, *Demetria*, *Rhodococcus*, *Janibacter*, *Leifsonia*, *Dermacoccus*, *Kocuria*, *Lapillicoccus*, *Microbacterium* and *Nocardioides* were present in soil samples from Antarctica (Figure 2) [56]. These isolates were tested against following pathogens: *C. albicans* ATCC 10231^T^, *S. aureus* ATCC 51650^T^, MRSA ATCC BAA-44^T^ and *P. aeruginosa* ATCC 10145^T^. Of all the present genera *Brevibacterium* showed the best antimicrobial activity, three isolates were active against *C. albicans*, one against MRSA and one against *S. aureus* [56].

Recently, thermal water sources have been explored in search for new antimicrobial entities. Analysis of the Tengchong hot springs revealed predominantly the presence of the genus *Streptomyces* (Figure 2) [57]. However, with the presence of *Actinomadura*, *Microbispora*, *Micromonospora*, *Nocardiopsis*, *Nonomuraea*, *Promicromonospora*, *Pseudonocardia* and *Verrucosispora*, a high number of rare non-*Streptomyces* actinobacteria could be found. Most of the isolates had a weak activity against *A. baumanii* and some against *M. luteus* [57]. Much stronger antimicrobial profile was exhibited by *Streptomyces* sp. Al-Dhabi-1, collected from Tharban hot spring in Saudi Arabia. Ethyl-acetate extract showed moderate to weak activity against tested pathogens, both Gram-positive and Gram-negative as well as fungi with MIC values <1 mg/mL [71]. Other recently explored habitats are cave systems populated with actinobacteria as its highly diverse microbial community may exhibit antagonistic properties against pathogens. Search for antifungal agents against *P. destructans*, causing white-nose syndrome in bats, led to the identification of 15 novel *Streptomyces* species, encouraging the further investigation of caves (Figure 2) [59] as valuable source for novel actinomycetes producing potent antimicrobials [59]. Unfortunately, most of the studies mentioned do not connect crude extract activity to known or novel natural products and are thus difficult to interpret regarding the potential for new antibiotics. Extreme environments may harbour many antibiotic natural products produced by a plethora of microorganisms, but solely investigate bioactivities without structural correlation to secondary metabolites is not beneficial for the discovery of potential new antibiotics.

### 2.2. Endophytic Actinomycetes

Endophytic actinomycetes represent a potential repository of novel bioactive compounds as there are almost 300,000 plant species, each hosting one or more types of endophyte [72]. Discovery of new compounds is however hindered by their slow growth and level of production, as it is usually significantly lower than the level of production of *Streptomyces* strains. The genus *Streptomyces* represents only 26% of all actinomycetes found in various plant tissues or in the rhizosphere. Cultured broth of 333 strains from plant roots and 137 strains from rhizospheric soil showed that isolated actinomycetes do have a potential as new sources of antimicrobials. Antimicrobial activity against *B. subtilis* KB-211 was present in 8.5% of isolates from plant roots, 9.1% against *K. rhizophila* KB-212, 5.7% against *X. campestris* pv. *oryzae* KB-88, 3.4% against *M. racemosus* KF-223, 1.7% against *C. albicans* and nothing is active against *E. coli* [73]. In general, the rhizosphere isolates are highlighting more isolates with antimicrobial activity. Overall, 20.3% of the rhizosphere isolates were active against *B. subtilis* KB-211, 17.1% were active against *K. rhizophila* KB-212, 8.9% were active against *X. campestris pv. oryzae* KB-88, 21.1% were active against *M. racemosus* KF-223, 9.8% were active against *C. albicans* and 8.1% were active against *E. coli* [73]. Bioprospecting rhizosphere isolates from the Caatinga biome (northeast Brazil) (Figure 2) [58] gave similar results as the above-mentioned example, where only 16% of isolates showed some activity against tested microorganisms. The most promising isolate was further investigated and crude ethanolic extract of *Streptomyces parvulus* D1.129 inhibited the growth of *S. aureus* and *B. subtilis* at 0.97 μg/mL. Despite the low percentage of isolates with antimicrobial properties, taxonomic difference and genome sequencing data strongly indicate the potential of these strains to produce novel bioactive compounds [58]*. Streptomyces* sp. strain RTd22—isolated from the Mexican sunflower *Tithonia diversifolia*—shows potential as a producer of novel natural products. Although analysis of the genome revealed an abundance of biosynthetic gene clusters (BGCs) not connected to known natural products, until now no novel bioactive compound was isolated from this strain [74].

### 2.3. Symbiotic Actinomycetes

**Figure 4:**
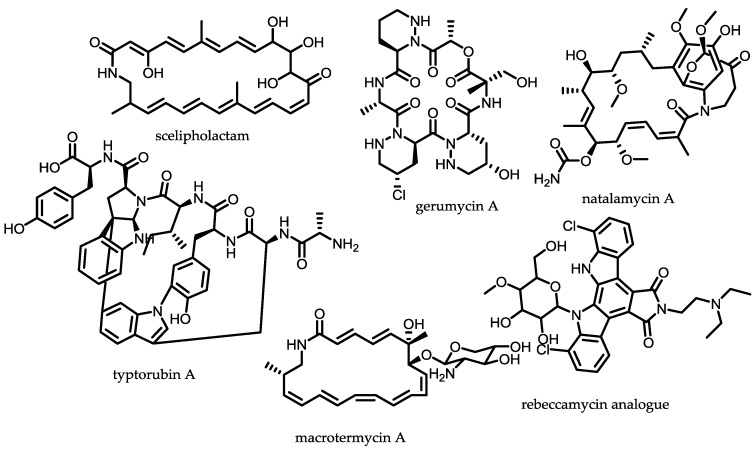
Novel natural products from symbiotic actinobacteria.

Bacteria and insects are ubiquitously distributed and some species have naturally developed a symbiotic relationship guided through a co-evolutionary adaptation, where the bacteria are located in specialised anatomical compartments. It is known that attine ants live in a tripartite mutualism with the fungi *Leucoagaricus gongylophorus*, which provides food to the ants, and with antibiotic-producing actinomycetes. Attine ants are hypothesised to acquire actinobacteria from the soil, selecting and maintaining those species that produce useful antibiotics as a way of biocontrolling the fungal counterparts [75]. Similar findings were discovered for wasp—*Streptomyces* and termite—*Streptomyces* symbiosis. Sceliphrolactam was found to be produced by a *Streptomyces* strain isolated from a wasp exoskeleton and inhibits the growth of amphotericin B resistant *C. albicans* [76] (Figure 4). A termite-associated actinomycete, *Amycolatopsis* sp. M39, produces glycosylated polyketide macrolactams, namely the macrotermycins. They exhibit very strong activity against *S. aureus* and *B. subtilis* and a moderate activity against *C. albicans* and *S. cerevisiae* [77]. Another termite-associated natural product, natalamycin A, exhibits a very high and broad-range antifungal activity [78]. *Pseudonocardia* spp. isolated from exoskeletons from several genera of ants were found to produce gerumycins, piperazic acid-containing cyclic depsipeptides that selectively inhibit a fungal pathogen *Escovopsis* [79]. A strain from *Pseudonocardia* spp. isolated from ant colonies harboured a plasmid encoding the biosynthetic gene cluster of a rebeccamycin analogue. Rebeccamycin was originally isolated from *Lechevalieria aerocolonigenes* (*Nocardia aerocolonigenes*) [80]. Rebeccamycin and several analogues have a very pronounced antitumour activity as they inhibit the mammalian topoisomerase I [81]. *Streptomyces* sp. CLI250 isolated from a fungus-growing ant yielded an unusual peptide tryptorubin A that harbours a few unique moieties, such as the linkage between two tryptophans in *para* position to the indole nitrogen. Another unusual structural feature is the linkage between tyrosine aromatic rings to the indole nitrogen of tryptophan, which feature has not been reported in any other natural product [82].

### 2.4. Conclusions

Underexplored habitats represent a largely untapped repository of compounds with unique chemical structures. Overall, new actinomycete isolates frequently exhibit moderate to strong activity against Gram-positive bacteria and fungi. Active compounds against Gram-negative bacteria remain a scarce finding in most habitats whereas the likelihood of finding anti-Gram-negative active natural products appears higher in rich, strongly diverse terrestrial communities. This is probably due to increased selective pressure as a consequence of the struggle of microbes to prevail in their fight for nutrients and territory in densely populated niches. In extreme environments, Nature itself already stamped out a large portion of the competition by making the habitat difficult to live in; therefore, it was previously assumed that the need of remaining microbes to utilise secondary metabolites is greatly decreased [83]. However, recent investigations propose that extremophile microorganisms are in fact able to produce diverse secondary metabolites [84]. The picture is admittedly incomplete since only few extremophiles have been systematically screened for the production of secondary metabolites; nevertheless findings from those that have been analysed are encouraging [84]. Access to products from extremophiles is also limited due to the lack of knowledge on how to isolate and cultivate them under laboratory conditions. As part of increased efforts to overcome this bottleneck, technologies such as the iChip platform can complement empirical improvements in isolation and fermentation of previously uncultured bacteria. Such novel microbiology-based methods are still underrepresented in most natural product discovery pipelines. It is worth taking into consideration that current discovery pipelines are more focused on exploiting new non-extreme habitats to obtain rare actinomycetes, which can be seen as a strategy to circumvent the need for extraordinary cultivation conditions. Although this approach cannot unlock access to extremophiles and uncultured microorganisms it is understandably the preferred approach implemented in many laboratories: as soon as microorganisms can be maintained under laboratory conditions, it is possible to make use of genome mining, apply genetic tools for the activation of silent biosynthetic gene clusters and scrutinise secondary metabolomes using a panel of sensitive analytical methods.

Taken together, access to the full complement of novel natural products within a single microorganism is only achievable through combination of available methods from multiple disciplines. Even without maintaining a constant supply of novel actinomycete isolates, these combined approaches can increase the availability of novel natural products. Considering that the majority of microorganisms cannot be cultured under laboratory conditions, the development of culture-independent approaches are indispensable before their metabolites can be investigated. Thus, eliminating the inherent bottleneck of cultivation is the distinguishing idea of metagenomic approaches, which are based on prior examination of biosynthetic gene clusters with *in silico* bioinformatics tools to enable heterologous expression of selected pathways originated from uncultured microorganisms. 

## 3. Metagenomic Approach to Exploit the Uncultured Bacterial Majority

Since to date only an estimated 1% of bacteria are cultured under laboratory conditions according to 16S ribosomal RNA (rRNA) [14], novel methods are required to access the greater diversity of natural products, circumventing the limitations of traditional culture-based approaches. Metagenomics as a culture-independent approach is based on DNA recovery directly from the environment (eDNA) to gain access to the hidden reservoir of secondary metabolite encoding sequences [85]. Metagenomic approaches in combination with classical culture-based approaches already became an important means to obtain deep insights into the microbial response to contamination or bioremediation techniques [86]. For example, microbial communities were used as environmental biosensors for nitrate [87] and uranium [87,88] or oil [89] contamination by probing 16S rRNA sequencing using high-throughput screening to determine the taxonomic composition of the microbial community. Since several key taxa are indicative of a particular contaminant, this metagenomic screening enables monitoring the presence and extent of contamination in the environment [86]. Therefore, this part of the review focusses on the recent developments in applied metagenomics employed in natural product research and aims to give an account of such culture-independent methods and involved technologies for accessing secondary metabolites from actinobacteria.

### 3.1. The Metagenomic Screening Workflow

**Figure 5:**
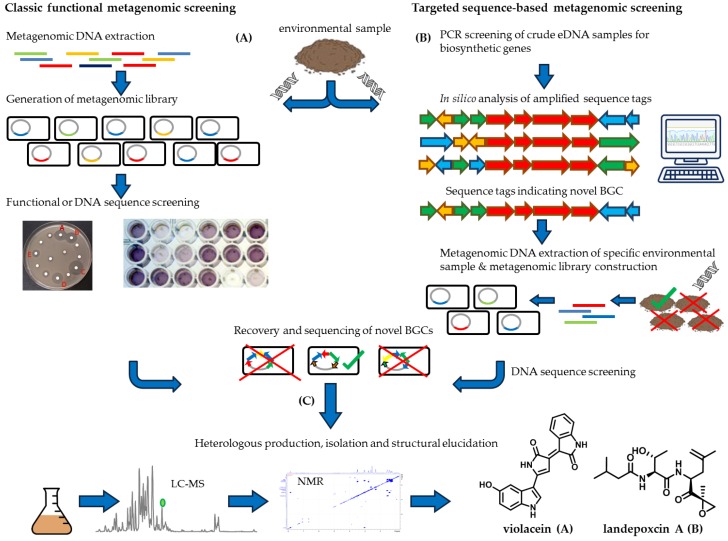
Scheme of metagenomic discovery pipelines adapted from Katz et al. (2016) [93]. Environmental samples are collected from ecologically and geographically diverse environments. (**A**) The classical functional metagenomic screening is based on the generation of metagenomic libraries by using appropriate heterologous hosts. Afterwards the metagenomic library is screened either for observable phenotypes or for the presence of target DNA sequence. (**B**) Crude eDNA is obtained from environmental sample and screened by PCR amplicons specific for sequences within biosynthetic machinery. DNA sequences tags are used to obtain *in silico* reassembled biosynthetic gene clusters (BGCs). A metagenomic library is generated from environmental samples harbouring the *in silico* reassembled BGC of interest. Environmental DNA (eDNA) is extracted and screened for the specific sequence of interest. (**C**) Novel BGCs are finally assembled and modified for heterologous expression in appropriate host and the produced natural product is isolated and structurally elucidated. Violacein is a natural product obtained by the approach (**A**) [91], and landepoxin is a natural product obtained by approach (**B**) [92].

In general, there are two main strategies applying metagenomic methods. The older strategy is relying on functional screening of individual eDNA clones and carried out with the help of huge cosmid libraries. Clones are typically selected by phenotypic readout such as visually detectable signals, bioactivity or more advanced screening procedures such as reporters/biosensors or through specific catalytic assays. The more recent strategy is based on biosynthetic considerations, relying on DNA sequence similarity of conserved biosynthetic genes to selectively get access to potential BGCs within the environmental sample. *In silico* selection of potentially interesting BGCs and subsequent heterologous expression can be used to finally access novel compounds [90]. Figure 5 summarises the main steps in the metagenomics workflow for both methods. Environmental samples are collected from ecologically and geographically diverse environments. In the classical functional metagenomic screening approach, metagenomic libraries are created by extracting, cloning and ligating environmental DNA (eDNA) into a shuttle vector and transformed subsequently into appropriate heterologous hosts. The metagenomic library is screened either for observable phenotypes or for the presence of target DNA sequence. Positive clones are recovered from the metagenomic library and the eDNA insert is sequenced. The antibacterial pigment violacein was isolated from soil metagenomic libraries by applying the classic functional metagenomic screening pipeline [91]. In the targeted sequence-based metagenomic screening approach, crude eDNA is obtained from environmental sample and screened by polymerase chain reaction (PCR amplicons specific for sequences within BGCs. DNA sequence tags are phylogenetically organised, evaluated for biosynthetic origin, compared to reference database and biosynthetic gene clusters are reassembled *in silico*. A metagenomic library is generated from environmental samples harbouring the *in silico* reassembled BGC of interest. Environmental DNA is extracted and screened for the specific sequence of interest. For both approaches, the novel BGCs are finally assembled and modified for heterologous expression in appropriate host and the produced natural product is isolated and structurally elucidated. An example of the targeted sequence-based metagenomic screening is shown by the proteasome inhibitor landepoxin A, which was discovered by comparing ketosynthase (KS) domain sequence tags to the KS domains from known epoxyketone biosynthetic gene clusters to identify epoxyketone proteasome inhibitor derivatives from metagenomic samples [92].

One of the major obstacles in the field of metagenomics is the usage of the model host organism *E. coli* with limited heterologous expression capabilities of complex natural products and the limited insert size of the cosmid-based libraries. These limited heterologous expression capabilities—in particular for secondary metabolites with corresponding huge modular biosynthetic architecture such as polyketides, nonribosomal peptides and hybrids of these—are not surprising, since *E. coli* is in contrast to actinobacteria and myxobacteria not a prolific producer of secondary metabolites [94]. Circumventing these major obstacles will be the key to improving novel natural products output from the metagenomics approach in the future. The usage of broad-host-range shuttle vectors allows effective eDNA clone shuttling to test heterologous expression in taxonomically diverse organisms [95]. Another approach to overcome limited heterologous expression capabilities of natural products in *E. coli* is to increase the transcription and translation of exogenous DNA by changing the host organism. Compared to *Streptomyces*, *E. coli* contains only half the number of different RNA polymerase sigma factor species. Furthermore, *Streptomyces* has been shown to express more genes deriving from metagenomic libraries than *E. coli* [96], revealing it as a potent host organism for heterologous expression. Because *E. coli* remains one of the few organisms that are featured with easy genetic manipulation as well as fast growth, additional measures than changing the host organism was taken into consideration to overcome its limitations. Overexpression of the missing sigma factor σ^54^ led to the production of oxytetracycline during heterologous expression of the previously silent oxytetracyline BGC originated from *Streptomyces rimosus* in *E. coli* [97]. This is just one powerful example demonstrating the utility of modified *E. coli* strains as heterologous platforms for metagenomic derived BGCs, indicating that even codon usage bias, starter and extender units availability are surmountable obstacles [98,99,100]. In particular, *E. coli* has proven to be an useful host for the heterologous expression of short biosynthetic pathways such as ribosomally synthesised and post-translationally modified peptides (RiPPs) [101], since the underlying biosynthetic machinery—the ribosome—is provided by the primary metabolism.

Even with a suitable broad host expression platform at hand, heterologous expression remains the critical part in the metagenomic workflow [102,103]. It is a common finding after reassembling of the whole BGC that most of the unmodified BGCs remain silent. To overcome this issue, several different non-targeted approaches are currently utilised such as simulation of environmental conditions through microcolony cultivation [104], co-cultivation [105] and the use of histone deacetylase (HDAC) inhibitors [106]. Targeted approaches to promote heterologous expression comprise the replacement of inactive native promoters through strong artificial or active native promoters, functional in the heterologous host either upstream of positive regulatory elements or individual biosynthetic genes. The operon structure of BGCs requires often the replacement of several promoters to enable heterologous expression; for example the anti-proliferative compounds lazarimide A and B deriving from a silent eDNA BGC, could only be expressed after yeast homologous recombination was employed to perform multiplex promoter exchange [107]. Another case study demonstrates that every gene from the eDNA derived BGC has to be controlled by a separate inducible promoter to finally produce erdasporine, a novel carboxy-indolocarbazole containing tryptophan dimer [108].

### 3.2. Direct Functional Metagenomic Screening

**Figure 6:**
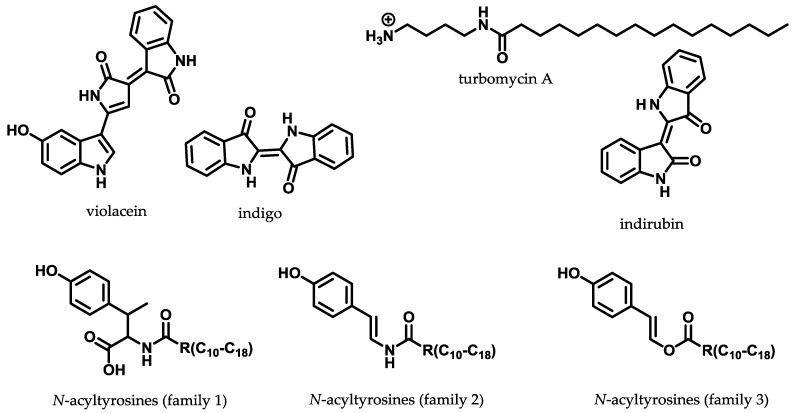
Example of natural products obtained by direct functional metagenomic screening.

Functional metagenomic screening is mainly but not exclusively based on phenotypic readouts such as visual detection and chromatographic separation, which is attributed to production of new small molecules. Another simple strategy is testing for growth inhibition against pathogenic microorganisms in a top agar overlay assay [109]. More advanced approaches use reporter/biosensor-based screens such as metabolite-regulated expression (METREX) and substrate-induced gene expression screening (SIGEX). METREX screening can be used to screen for production of quorum-sensing inhibitors able to bind to the LuxR transcriptional activator, which induces expression of a target gene. This leads to accumulation of a green fluorescent protein (GFP) used as reporting system for positive cosmid clones inducing bacterial quorum sensing [110]. SIGEX is based on the substrate-induced expression of a catabolic gene with the similar reporting system for positive cosmid clones such as METREX [111]. Both methods can be used for discovering new genes and gene products that would not have been detected with a sequence-based approach, since no prior knowledge of the gene and gene product is necessary. However, the outcome of natural products discovered by functional metagenomics is to date rather disappointing with only few successful examples such as the antibacterial pigments violacein [91], indirubin with its isomer indigo [112], *N*-acyltyrosines [113] and the turbomycins [114] as shown in Figure 6. All of these examples show simple non-actinomycte derived secondary metabolites, synthesised by very few biosynthetic enzymes. Hence, functional metagenomic screening is obviously failing to access the complexity of actinomycete secondary metabolism.

Enzymatic activity unique or predominantly present in secondary metabolism can be used for advanced functional phenotypic screening to improve the identification of functional BGCs [115,116]. Owen et al. [117] described for example a screening approach based on the single module nonribosomal peptide synthetase (NRPS) *bps*A as reporter gene that synthesises the coloured indigoidine from L-glutamine through activation by 4-phosphopantetheinyl transferase (PPTase)-containing clones. *PPTase* genes are often located in close proximity to a BGC, however most of the BGCs are remote from their required *PPTase* gene, since most microorganisms possess only one *PPTase* gene in their genome, which is involved in secondary metabolism [118]. Wherefore indigoidine production in general is a powerful indicator to identify positive eDNA clones containing BGCs deriving from NRPS or polyketide synthase (PKS) pathway machinery. Five years after inventing this functional *bpsA* gene expression-type BGC screening method, Brady and co-workers screened a soil eDNA library hosted in *S. albus::bpsA ΔPPTase* and identified clones containing NRPS, PKS and NRPS-PKS biosynthetic gene clusters, resulting in the rediscovery of myxochelin A the biosynthesis of which is dependent on a very simple NRPS [119]. This study is a proof of concept for an innovative functional screening method albeit it suffers from a rediscovery issue based on the DNA-sequence paralleling the traditional culture-based high rediscovery rate based on the metabolic level. Furthermore, the relatively simple structure with its underlying plausible biosynthetic pathway, consisting of four biosynthetic proteins (with only one NRPS module) [120], is questioning the general applicability of this method for more complex biosynthetic pathways, since many BGCs are exceeding the typical insert size of a cosmid preventing the functional detection.

### 3.3. Sequence-Based Metagenomic Discovery Efforts

In contrast to functional metagenomic screening, the identification of potential BGCs through the sequence-based metagenomics approach relies on *in silico* analysis of sequence tags representing microbial genomes for the initial screening process instead of attempting heterologous expression. The complex mixtures of PCR amplicons consisting of numerous domain fragments from potential biosynthetic gene clusters deriving from environmental samples are defining the term of Natural Product Sequence Tags (NPSTs). Unlike whole genome sequences, those datasets are consisting of much shorter DNA stretches, which hampers the identification of biosynthetic gene clusters. Therefore, conventional tools in the field of natural product research such as antiSMASH [121,122] are less suitable for analysing those datasets. Predictive tools such as Surveyor of Natural Product Diversity (eSNAPD) [123] and Natural Product Domain Search (NaPDos) [124] are based on the phylogenetic relationships of sequence tags which enables searching for both close and distant relatives in the large datasets [125]. The advantage of eSNAPD to organise NPST is the requirement of little computational power and the comparable low cost of sequencing small PCR amplicons in contrast to whole genomes. One nuisance with sequence-based metagenomic discovery is the enormous size and density of soil metagenomes in soil samples, where a single sample can contain up to 10^5^ unique genomes [93]. Furthermore, metagenomic DNA libraries are sparsely populated with biosynthetic genes of interest, since only a fractional amount of each genome is devoted to secondary metabolism [126]. Therefore, gene cluster enrichment strategies can be used to simultaneously reduce the size and increase the density of biosynthetic sequence tags within eDNA libraries. Employing PCR in sequence-based approaches using degenerated PCR primers to amplify biosynthetic genes of interest was shown to be more sensitive in identifying variants of biosynthetic domain sequences with known architecture than shotgun sequencing [127]. However, these amplicon sequencing-based methods are inherently biased, with regards to detect standard gene organisation such as RiPPs, hybrid NRPS-PKS and nucleoside antibiotic sequences [101,128]. In general, modern sequence-based methods are centred on amplifying conserved biosynthetic enzymes via degenerated primers to construct metagenomic libraries rather than analyse randomly crude eDNA since the majority of DNA sequences are not associated with biosynthetic pathways. A promising conserved biosynthetic gene utilised to find novel metagenomic-based natural products is for example the KS-β [129]. Identification of tailoring and other enzymes occasionally involved in biosynthesis of natural products such as sulfotransferases [130], esterases [131], lipases [132], β-galactosidases [133] and isonitrile synthases [134] could also be utilised to obtain novel natural products such as new sulfated glycopeptide derivatives [130] or (*E*)-3-(2-isocyanovinyl)-1*H*-indole [134] from metagenomic samples. However, those specialised screening approaches of individual biosynthetic sequences are not offering a fully systematic method for accessing the complex entirety of available metagenomes, since these enzymes are also distributed in primary metabolism, and are not common for secondary metabolism, in particular esterases and lipases.

**Figure 7:**
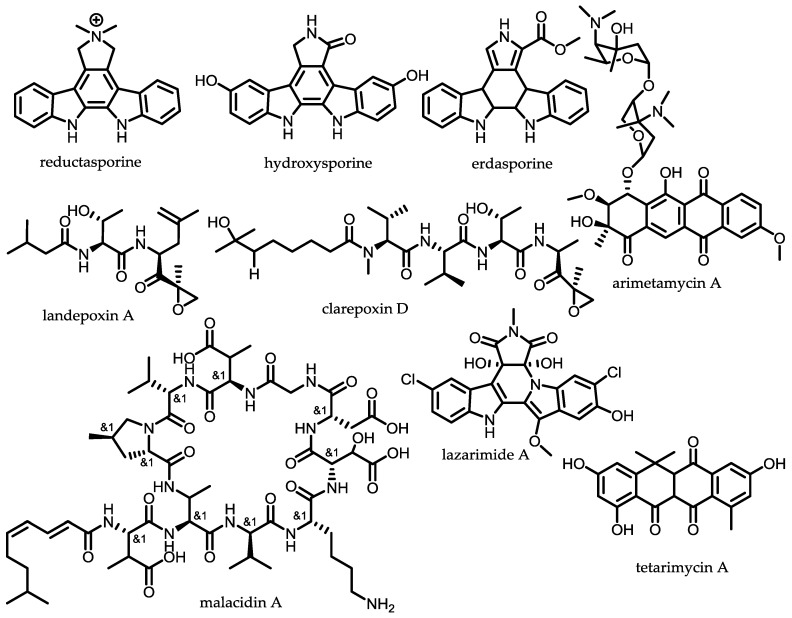
Example of natural products obtained by sequence-based metagenomic approaches.

Brady and co-workers could obtain the rare tryptophan dimers hydroxysporine and reductasporine [135], the cytotoxic anthracycline arimetamycin A [136], the tetracyclic MRSA-active antibiotic tetarimycin A [137], the antibiotic cyclic lipopeptides malacidins A and B [138] and the epoxyketone proteasome inhibitors clarepoxcins A–E and landepoxcins A and B [92] with the sequence-based metagenomic approach (Figure 7). These natural products were obtained by using *Streptomyces albus* as heterologous host, underlining this strain as a gifted heterologous host in particular for actinomycete derived BGCs [139]. Recent developments of utilising different so-called broad host heterologous expression platforms for microbial natural product biosynthetic pathways—in particular various hosts from different phylogenetic background such as myxobacterial microorganisms—was described by Müller et al. [102]. The calcium-dependent antibiotics malacidins A and B were discovered by using degenerated primers targeting NRPS adenylation domains to generate amplicons from an arrayed collection of eDNA isolated from 2000 unique soil samples. The malacidins are structurally distinct in comparison to known calcium-dependent antibiotics such as daptomycin and friulimicin, which emphasises the sequence-guided metagenomic pipeline to probe for new congeners of the natural product family. In contrast, the tryptophan dimers were discovered by screening diverse soil samples using degenerate primers for the presence of unique chromopyrrolic acid synthase (CPAS) sequences. This approach demonstrates the large-scale sequence tag-based pre-filtering of diverse environmental samples as input for the metagenomic pipeline to push the discovery of novel BGCs [135]. It is also possible to use NPST libraries to recover natural product genes from metagenomic libraries to obtain through the unique sequence label (barcode) the BGC of interest and the specific sub pool of the library [125].

However, many BGCs are exceeding the typical insert size of a cosmid, and thus several overlapping DNA cosmid clones spanning the complete biosynthetic gene cluster have to be reassembled into a bacterial artificial chromosome (BAC) by the frequently used method of transformation-associated recombination (TAR). Afterwards, the reassembled BGC can be transferred from yeast into different bacterial strains for heterologous expression. Consequently, this approach is rather a heterologous expression based on a metagenomic library than an expression of metagenomic samples [140].

### 3.4. Metagenomics for the Assessment of Marine Endophytes

**Figure 8:**
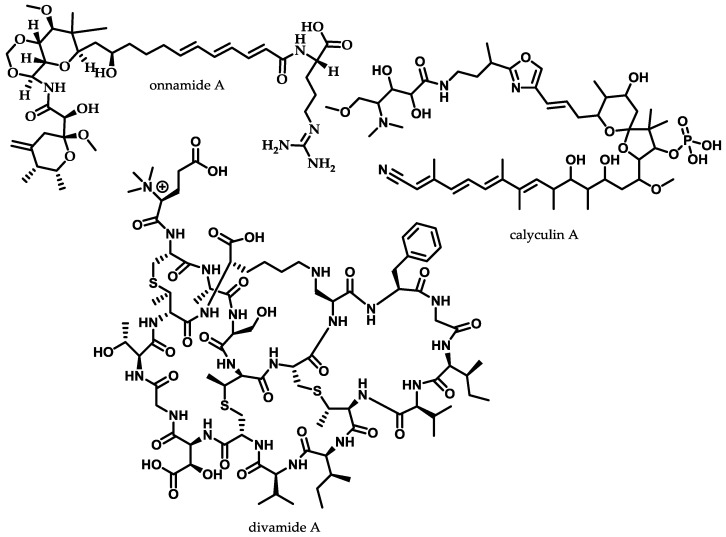
Example of marine natural products with elucidated biosynthesis achieved through metagenomic approach.

Besides the extensively investigated ecology of soil-dwelling bacteria, the marine environment is still less investigated in terms of natural product research (see Section 2.1) [12]. The major drawback of marine natural product discovery is that cultivation of bacteria is frequently challenging and production of compounds of interest is often not possible in sufficient amounts for structure elucidation [141]. Occasionally, serendipitous discovery of alternative producers of marine natural products might enhance the production and help to characterise subsequently the underlying biosynthetic pathway. One example are the bengamides, a class of natural products that have been characterised as inhibitors of methionine aminopeptidases, emphasising the potential as anticancer compounds [142]. Previously, they have been assumed to exclusively derive from sponge *Jaspis* cf. *coraciae* until they have been found in the terrestrial myxobacterium *Myxococcus virescens* ST200611 (DSM 15898) which allowed studies to be conducted on the biosynthesis of bengamides, their heterologous expression, and the self-resistance mechanism of their producer [143,144]. However when no alternative producer can be found, metagenomic approaches are playing a crucial role to access the diversity of marine natural products by identifying biosynthetic gene clusters for the heterologous production of secondary metabolites [109]. This approach can provide information about the origin of biosynthetic gene clusters, as compounds thought to be produced by sponges, have been later on elucidated to originate from their bacterial symbionts [145]. One example are the unusual antifungal peptides microsclerodermins, which have been reported to derive exclusively from the marine genera *Microscleroderma* and *Theonella* [146,147]. Recent findings, however, revealed the terrestrial alternative producers *Jahnella* sp. MSr9139 and *Sorangium cellulosum* So ce 38 producing microsclerodermins and pedeins along with additional derivatives [148]. The genomic sequence for these two myxobacterial producers enabled to propose a conclusive biosynthetic model for the underlining pathway. Combined with recent metagenomic studies emphasising myxobacteria as putative sponge symbionts, these outcomes provide evidence that a terrestrial bacterial symbiont might be the real biosynthetic source of this “marine” natural product [149]. Considering that seawater bacteria are presumably 10-fold less represented in cultured isolates compared to soil-dwelling bacteria [150], culture-independent methods in marine environment could be expected to access a larger untapped reservoir of chemical and biological diversity of natural products than soil-dwelling bacteria. The following section briefly highlights recently conducted metagenomic case studies, which were guided by previously characterised structure or associated biological activity. The first and second example describes the biosynthetic characterisation through metagenomics of the endosymbiotic derived polytheonamides and calyculin A, the last case study highlights the recent discovery of the anti-HIV lanthipeptides, the divamides, which exemplifies the convergence of traditional functional screening and sequence-based metagenomics (Figure 8).

The marine sponge *T. swinhoei* contains numerous uncultivated bacterial symbionts and is a prolific source of bioactive secondary metabolites [151]. One of those marine natural products with remarkable structure is the 48-residue large peptide polytheonamide, incorporating 13 nonproteinogenic amino acids. Therefore, it was assumed for years that the marine cytotoxic polytheonamide originates from a nonribosomal peptide biosynthesis, despite the gigantic size of 48-residue NRPS machinery [152]. To clarify the biosynthetic origin of the polytheonamides, a semi-nested PCR approach with designed primers specific for precursor peptide consisting of proteinogenic l-configured amino acids was conducted from *T. swinhoei* metagenomic sample [153]. The assembled sequenced amplicons revealed 11 clustered genes, with seven open reading frames forming an operon. Furthermore, several bacterial transposition elements and Shine–Dalgarno sequences were found; these findings not only confirm the ribosomal origin but also suggest that the polytheonamides are produced by bacterial endosymbionts [154]. Further studies of the sponge *T. swinhoei* involving single-cell genomics assisted by enriched bacterial fractionation, fluorescence assisted cell sorting and whole genome amplification combined with pathway specific PCR, revealed the *Entotheonella* spp. bacterial symbiont as the native producer. Similarly, *Candidatus Entotheonella* spp. was assigned as the non-cultured bacterial symbiont of *T. swinhoei*, responsible for the production of onnamides [155] (Figure 8).

Calyculin A first isolated from the marine sponge *Discodermia calyx* in 1986, is a PKS-NRPS hybrid cytotoxic natural product [156]. Since further calyculin-related natural products have been isolated from different sponges, it was assumed that associated bacterial symbionts are producing those compounds [157]. Recently the biosynthetic gene cluster was identified by a metagenomic approach comparable to the methods described for the identification of the BGCs of onnamides and polytheonamides. The PKS-NRPS hybrid origin of calyculin leads to the design of primer screening trans-acyl transferase (AT)-type KS, adenylation domains and 3-hydroxy-3-methylglutaryl-coenzyme A synthase (HMGS)-like motifs using *D. calyx* metagenomic DNA as a template. In total 250,000 clones of a metagenomic library of *D. calyx* total DNA had to be controlled applying a sophisticated pooling strategy [158], before the calyculin BGC could be assigned [159]. Furthermore, the biosynthetic gene cluster was used as a probe, employed catalysed reporter deposition-fluorescence *in situ* hybridisation (CARD-FISH) and laser microdissection was revealing a filamentous bacterium with 97% identity in the 16S rRNA sequence to the *Candidatus Entotheonella* symbiont from the *T. swinhoei* sponges.

Organic extract of the Prochloron-harbouring tunicate *Didemnum molle* (E11-036) from the Eastern Field of Papua New Guinea represented a promising hit in an anti-HIV assay. However further assay-guided fractionation and spectroscopic analysis could not reveal the complete chemical structure of the natural product responsible for the observed antiviral activity, due the very limited sample size. The natural product could at least preliminarily be determined as a novel peptide containing the modified amino acids lanthionine and *N*-trimethylglutamate, as well as the partial peptide sequence “GTTR”. The acquired assembled metagenome was searched for the amino acid motif GTTR and for overrepresentation of thioether cross-linked amino acids (cysteine, threonine and serine at the C-terminal end) from which lanthipeptides derive their name [160], yielding one putative divamide BGC. Combined *in silico* analysis of the biosynthetic pathway and re-examination of the acquired NMR (nuclear magnetic resonance spectroscopy) data culminated in the structure elucidation of the divamides. Moreover, heterologous expression of different divamide BGCs originating from the symbiotic cyanobacteria *Prochloron didemni* in *E. coli* confirmed the predicted structure and the biosynthetic pathway [161]. The discovery of the divamides highlights the synergy of different strategies for the search of novel bioactive natural products whereas biological activity within an ecologically relevant system is screened first by functional screening, with the resulting chemical and metagenomic approaches following promising biology. The scarcity of isolated material is circumvented by applying NMR structure elucidation combined with metagenomics and synthetic biology to fully characterise newly discovered natural products and their underlying biosynthesis.

The last case study demonstrates the usage and value of metagenomic approaches in terms of biosynthetic gene cluster identification for the discovery of new natural products from marine endophytes. The existing examples to date have afforded mainly PKS, NRPS or PKS-NRPS hybrids, assembled by gigantic modular biosynthetic pathway machineries. The high mutual similarity of corresponding domain types, in particular KS and AT domains in PKS and condensation (C) and adenylation (A) domains in NRPS, greatly facilitates the *in silico* identification of genes encoding these gigantic microbial biosynthetic pathway machineries, as long as they follow the so-called collinearity rule [162]. In contrast, other types of biosynthetic pathways such as RiPP and nucleoside antibiotics may be much smaller and less obviously clustered than multifunctional NRPS and PKS gene clusters and thus more difficult to identify. However, in terms of heterologous expression, the smaller RiPP BGCs are much easier to express than PKS, NRPS or PKS-NRPS hybrids with the underlying advanced biosynthetic machinery. In terms of structural features, they nevertheless may be at least equally interesting. Additionally, the biosynthetic characterisation of the polytheonamides is demonstrating the use of modern single cell separation and sequencing technologies, which might accelerate the future perspective of metagenomic approaches.

### 3.5. Sequence Boom: Potential of Next Generation Sequencing and Single-Cell Genomics

With affordable sequencing costs for whole genome sequencing, it became obvious that genome sequencing of single cells would give new perspectives of genetic analysis. In the field of metagenomics, single-cell microorganism sequencing enables genome assembly of new phyla and grant new insights into the microbial dark matter as it can be used for the “non-culturable” bacteria [163], such as described for *T. swinhoei* above. Furthermore, single-cell genomics provide a valuable advantage by reducing the complexity of the genomic signal through the physical separation of cells or chromosomes. However, obtaining the whole genome from a single DNA molecule harboured by individually isolated cells is a technically challenging procedure. Especially the guanine-cytosine (GC) rich genomes of actinobacteria and myxobacteria, both prolific sources of natural products [164,165], are emphasising the problems of whole genome sequencing techniques relying on short reads (typically in the range of several hundred base pairs) [166]. The four major technical challenges in the field of single-cell genomics are efficient isolation of individual cells, amplification of the genome of the specific cell to circumvent the scarcity of genomic material, sequencing of the previously amplified DNA amplicons representing the genome sequence and the evaluation of acquired data [163]. Three whole genome amplification (WGA) methods are commonly applied now: pure PCR-based methods such as degenerate oligonucleotide primed PCR (DOP-PCR), isothermal based methods such as multiple displacement amplification (MDA) and hybrid methods of both such as multiple annealing and looping based amplification cycles (MALBAC) and PicoPLEX. PicoPLEX is based on an initial isothermal preamplification followed by PCR amplification of the amplicons generated during the first step. Among the WGA methods, DOP-PCR achieves higher coverage uniformity with a low physical coverage of the genome. MDA on the contrary exhibits greater genome coverage with lower coverage uniformity of the genome and the hybrid methods MALBAC and PicoPLEX are characterised by coverage and uniformity [163]. A convergent method approach of single-cell genomic sequencing based on MDA and metagenomic library screening accelerated the identification of the apratoxin A biosynthetic gene cluster, a potent cytotoxic compound [167]. Furthermore, the genomes of two chemically distinct *Entotheonella* symbionts, the producers of onnamides and polytheonamides, could be reassembled by using combined single cell isolation and MDA based whole genome amplification [155].

Nevertheless, a truly novel natural product was not discovered to date using single-cell isolation combined with MDA-based whole genome amplification. Single-cell genomics has the potential to assemble the genomes of species that are present at low frequencies in metagenomic samples [167], as well as to produce assemblies of genomes of completely uncharacterised microorganisms. In conclusion, this interesting technology is highlighting great promises and single-cell genomic sequencing might advance the field of natural product research, however the downstream limitations of the metagenomic workflow remain challenging for the discovery of novel compounds.

### 3.6. Conclusions and Future Considerations

Since metagenomics is a relatively new technology, further advances are necessary to overcome major inherent bottlenecks not only for functional but also for sequence-guided approaches. Brady and co-workers could uncover novel natural products such as the calcium-dependent antibiotics malacidins A and B or the epoxyketone proteasome inhibitors clarepoxcins and landepoxcins via metagenomic approaches. In the field of marine natural product research, metagenomic approaches have been used to identify the biosynthetic pathways of pharmaceutically relevant compounds. Nevertheless, both approaches could not have been successfully conducted without fundamental biosynthetic knowledge. Besides the successful discovery of novel natural products, there are still some key limitations which have to be circumvented in the future [168]. One of the main bottlenecks of metagenomics is the ability to isolate DNA from soil sample for the generation of metagenomics libraries, since samples are inhabited by a great variety of microorganisms and collecting samples containing eDNA harbouring novel biosynthetic pathways is time consuming and requires fundamental ecological background knowledge. Substantial improvement has already been achieved in terms of extraction of high molecular weight DNA and elimination of environmental inhibitors by synchronous coefficient of drag alteration (SCODA) [169,170], indirect DNA extraction through microbial cell separation and formamide treatment [171]. Another limitation arises from the heterogeneous origin of metagenomic libraries, which prevents the taxonomical correlation of biosynthetic gene cluster or bioactivity to the corresponding microorganism. Managing the size and complexity of metagenomic datasets will require for the long-term automated genome mining tools and pattern recognition based algorithms [172]. Nevertheless, these data processing obstacles might be overcome in the near future, since the field of bioinformatics is evolving rapidly; therefore, the final heterologous expression of (reassembled) BGCs remains the major limitation in the near future. Very often promising biosynthetic gene clusters functional in the natural producers remain silent in the heterologous host. Different codon usage, rare tRNAs, tightly regulated promoters, toxicity and stability are only a few of the diverse obstacles. Expressing BGCs from underrepresented bacterial taxonomic origin is still challenging, since even so-called broad host heterologous expression platforms are not always capable of producing the corresponding natural products. Therefore, further progress in understanding the biosynthetic logic of assembly lines found by metagenome mining, including ways to achieve heterologous expression, will help to overcome this inherent bottleneck.

## 4. Genome Mining: Current Reality and Future Promise of the Post-Genomic Era

With first whole genome sequence of the model microorganism *Streptomyces coelicolor* A3 (2) [13] published in 2002, it became evident that actually little was known about the so-called well-studied strain in terms of secondary metabolism. By that time, *S. coelicolor* was known to produce actinorhodin, methylenomycin, calcium-dependent antibiotic and undecylprodigiosin [173,174,175,176]. The last of these compounds was discovered already in 1985. Development of antiSMASH [177] in 2011, by now the most commonly used tool for automatic genomic identification and analysis of biosynthetic gene clusters [121,178], allowed the prediction of additional BGCs located in the genome of *S. coelicolor*, indicating potential for production of hitherto unseen metabolites. This section therefore gives an overview about how biosynthetic gene clusters can be accessed for the discovery of novel natural products. Besides prediction of biosynthetic gene clusters and possible read-outs of *in silico* analysis, the impact of genetically minimised strains for heterologous expression and different approaches to target silent gene clusters will be discussed in detail.

### 4.1. Biosynthetic Gene Cluster Prediction and Targeted Activation of BGCs

Every experimental method for the assessment of a biosynthetic gene cluster relies on *in silico* analysis of the bacterial genome. NRPS-precursors and modular polyketide synthases assembly lines can be predicted by NRPSpredictor2 [179] or the Stachelhaus code [180], by domain organisation and their predicted substrate specificity [181], respectively, both algorithms being integrated into antiSMASH. Bioinformatic prediction of not yet seen natural product structures based on biosynthetic genes is a powerful tool for follow-up analysis, such as genome–metabolome correlation. However, structural prediction of natural products solely based on *in silico* data is still not reliable. Targeted activation or knockout of BGCs requires bioinformatic analysis to prioritise the selection of the BGC. Natural products with structures from new compound families are favoured, therefore online tools that correlate biosynthetic gene clusters of NRPS and RiPPs with analytically acquired data are developed [182,183] as described in more detail in Section 5.2. BGCs are also being analysed for resistance genes located close to the biosynthetic genes that could provide information about the mode of action of the associated natural product. Predictions are available as an online tool PRISM3 [184,185] and recently developed the Antibiotic Resistant Target Seeker (ARTS) [186]. Cases of resistance genes located within the biosynthetic gene cluster are reported, for example, oxytetracycline with *otrA* gene encoding the elongation factors EF-Tu and EF-G that protect ribosomes of the producer strain [187] and griselimycin’s *griR* resistance gene encoding an additional copy of the DNA polymerase III beta subunit [188]. Therefore, the prioritisation of BGCs according to the resistance genes found in the gene cluster shows great promise specifically to identify novel antibiotics. Beside these specialised self-resistance conferring genes, there are more general resistance genes located within BGCs; during the heterologous expression of bottromycins in *S. coelicolor*, the replacement of the native promoter by the strong ermE* promoter in front of the gene encoding the respective efflux pump *botT*, showed a 20-fold increased production concentration compared to the natively expressed resistance gene [189].

### 4.2. Utilising the Complexity of the Biosynthetic Machinery for the Discovery of Novel Natural Products

**Figure 9:**
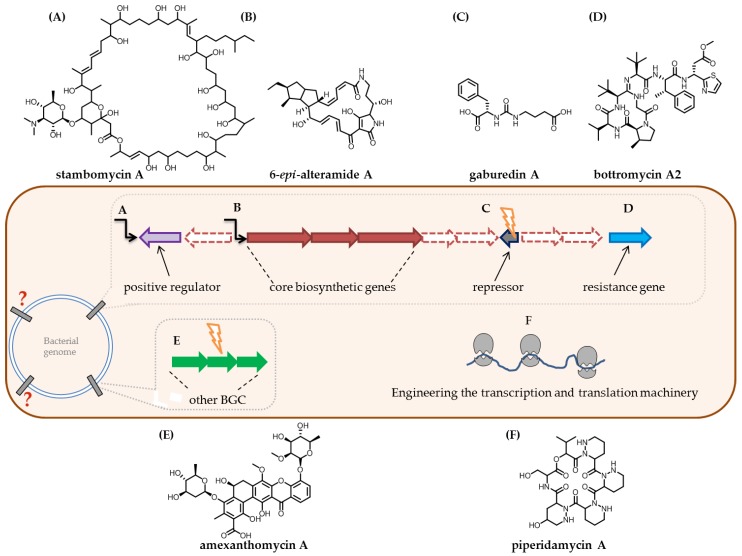
Generic representation of BGC consisting of core biosynthetic genes, genes for tailoring enzymes (dashed line), positive and negative regulators and self-resistance conferring genes. Strategies can be employed in the native or heterologous host. (**A**) A promoter is either inserted in front of a positive regulator or an additional copy of the positive regulator with a promoter is inserted in the genome; consequently, a higher concentration of natural product is expected. The macrolide stambomycin A was obtained through constitutive expression of an LAL family regulator [197]. (**B**) Promoter is inserted in front of the operon of biosynthetic genes enhancing transcription and production of natural product. The polycyclic tetramate macrolactam 6-*epi*-alteramide A was obtained by introducing the ermE* promoter in front of the hybrid type I PKS-NRPS operon [198]. (**C**) Repressor gene is disrupted, production of natural product is enhanced. Inactivation of the repressor *gbn*R in *S. venezuelae* induced the production of gaburedin A [196]. (**D**) Resistance gene protects producer strain against its own natural product. During the heterologous expression of bottromycins in *S. coelicolor*, the replacement of the native promoter by the strong ermE* promoter in front of the efflux pump *bot*T, showed a 20-fold increased production concentration compared to the natively expressed resistance gene [189]. (**E**) In some cases, knocking out the biosynthetic genes of known compounds in producer strains enhances biosynthesis of other encoded natural products in the genome. Amexanthomycin A was found after knocking out PKS *rifA* gene responsible for rifampicin biosynthesis from *Amycolatopsis mediterranei* S699 [190]. (**F**) Targeted induction of mutation in RNA polymerase and ribosomal proteins by antibiotics can cause upregulation of BGC expression. Mutations in *rpsL* and *rpoB* genes activated the silent BGC of piperidamycins from *S. mauvecolor*, culminating in the isolation of the antibacterial piperidamycin A [209].

Genetic modification of producer strains of interesting natural products is often challenging, as they are featured with several BGCs and manipulation often affects more than one metabolic pathway. In some cases, knocking out one biosynthetic gene of a known compound in a producer strain can therefore enhance the biosynthesis of other encoded natural products in the genome. The newly discovered ten pentangular polyphenols, namely amexanthomycins A–J [190], were only found after knocking out the *rifA* PKS gene responsible for rifampicin biosynthesis from *Amycolatopsis mediterranei* S699 (Figure 9).

In addition, transcription regulators encoded nearby the biosynthetic gene clusters of known natural products have a great influence on secondary metabolite expression [191,192,193]. Some act as activators and some as repressors, but exchange of their natural promoters with constitutive or inducible promoters can lead to overexpression or inhibition of the production of targeted BGC. For example the null mutation of the *tetR* repressor within two silent BGCs in *Streptomyces* sp. PGA64 and *S. ambofaciens* culminated in the isolation of the novel angucyclinone metabolite UVM6 [194] and the previously described kinamycins [195], whereas the production of gaburedin A was induced through the inactivation of the repressor *gbnR* in *S. venezuelae* [196]. In contrast, the discovery of stambomycins from *S. ambofaciens* was achieved by constitutive expression of a LAL family regulator, acting as activator of the stambomycin biosynthesis [197]. Exchange of the native promoter with an inducible promoter, upstream of the first biosynthetic gene in the operon, which can be tightly regulated, provides control over biosynthetic genes. For example, the polycyclic tetramate macrolactam 6-*epi*-alteramide A was obtained by introducing the strong ermE* promoter in front of the hybrid type I PKS-NRPS operon [198] (Figure 9). Without induction no metabolite can be detected, comparable to a knockout mutant, but addition of inducer promotes metabolite overexpression [199]. For the above-mentioned promoter exchange strategies, decent understanding of operon structures is required in combination with comprehensive metabolic profiling with accurate detection systems such as high-resolution mass spectrometry to detect small concentrations and corresponding the target masses. Currently-used native, modified and synthetic promoters were summarised by Rebets et al. [191]. As promoters might be necessary for yield improvement, research groups are working on expanding promoter libraries and they already reported the successful generation of 56 synthetic promoters. Three of these promoters of different strengths are active in multiple actinobacterial strains [200] and 38 synthetic promoters ranging from weak, medium to strong promoters are active in several *Streptomyces* species [201]. This finding opens the potential for much better control of genetic regulation in the future. This is of importance as highest expression often does not go hand in hand with increase of product yield due to limitations in translational machinery or self-resistance, just to name a few.

Considering this observation, the generation of strains with minimalised genomes is also crucial to have a heterologous expression platform where gene functions can be studied in detail and metabolic changes can easily be correlated to specific manipulations in the strain. For actinomycetes several strains with minimalised genomes are available such as *S. albus* Del1, *S. albus* J1074 and *S. avermitilis* SUKA17 [202,203]. Unnecessary genomic parts, such as insertion sequence (IS) elements and secondary metabolite gene clusters, are removed to increase genetic stability and reduce the metabolic burden. *S. coelicolor* M1152 with four deleted native biosynthetic gene clusters and additional point mutations introduced into *rpoB* and *rpsL* genes enhanced transcription and translation, respectively; depending on the heterologous cluster type, the obtained strain produced 20–40 times more of the compounds under study when compared with parental *S. coelicolor* M145 [204]. After successful implementation of such platforms with minimalised genomes, they can be used for the expression of silent BGCs from various strains and heterologous expression of BGCs found with metagenomic platforms.

### 4.3. Silent BGC Activation by Chemical Elicitors, Ribosome Engineering and Chromatin Remodelling

Many BGCs found in genome sequence that do not have detectable natural product, due to small concentrations being produced under certain growth conditions, probably promoting negative regulation, are termed silent or cryptic biosynthetic gene clusters [205,206]. To access “silent” BGCs there are two strategies currently applied. Either they are targeted by empirical optimisation of growth conditions [206], addition of chemical elicitors [106,207], trace metal ions [208], provision of exogenous small molecules to producer strain [205], ribosome engineering [209,210,211] or using targeted approaches based on genome sequence [191] with some of them mainly focusing regulatory genes [193]. Empirical variation in growth conditions such as temperature, pH, co-cultivation, or addition of chemical elicitors can induce BGC expression. Natural products such as goadsporin promotes secondary metabolism and morphogenesis in *Streptomyces* [212], subinhibitory concentrations of trimethoprim activated the expression of the malleilactone BGC from *Burkholderia thailandensis*—silent under standard laboratory conditions [213]—siderophore desferrioxamine E produced by *S. griseus* stimulates growth and development of *S. tanashiensis* in co-cultivation experiments [214]. In 2016, a Canadian compound collection of 30,569 small molecules was screened for their ability to alter the pigmentation of *S*. *coelicolor* colonies, usually linked with production of different secondary metabolites, during growth on solid medium. Several compounds referred as ARC2 served as a potent general elicitor inducing production of cryptic metabolites [215]. Its derivative Cl-ARC was used on 50 different actinomycete strains; 10 μM Cl-ARC was added to five growth solid media per bacteria and bacteria were grown for seven days at 30 °C. *N*-butanol extracts of controls and Cl-ACR treated bacteria were subjected to comparative LC-MS analysis. At least 23% of BGCs got activated in addition to three rare secondary metabolites that showed activity against bacteria and/or against eukaryotes [216]. However, follow-up studies to show that indeed novel metabolites are produced at larger scale have yet to be reported.

Targeted mutation induction in RNA polymerase and ribosomal proteins by antibiotics rifamycin, streptomycin, and gentamicin can cause upregulation of BGC expression. Mutations in *rpsL* and *rpoB* genes activated silent BGC of piperidamycins from *S. mauvecolor* [209] and are shown to awaken cryptic BGCs [210] (Figure 9). Chromatin remodelling approaches were used on fungi, but interestingly these approaches were also applicable on *Streptomyces* species [106,205] as bacterial genomes are compacted presumably by nucleoid-associated proteins, RNAs and differential supercoiling leading to a comparable compaction for certain genes such as in eukaryotic organisms [217]. Cryptic BGCs could be located in tightly packed heterochromosome regions therefore chromatin remodelling or epigenetic modifications with addition of DNA methyltransferase (DNMT) and histone deacetylase (HDAC) can influence their expression [218]. Up to date, no new natural products were found applying these approaches, but differences in biosynthetic gene expression were observed even though in majority of cases expression was found reduced.

### 4.4. Conclusions

The majority of the above mentioned empirical methods or targeted approaches yielded examples for the identification of new natural products. Development of bioinformatic prediction tools improved genome mining and prioritisation of BGC, however there is definitely a lack of standardisation. New genomes are sequenced and prediction tools are being developed and provide useful platform although they lack the ability to perfectly predict compound structures. Even though biosynthetic gene cluster prediction algorithms are available, *in vivo* or *in vitro* confirmation are obviously the only way forward. Deciphering of biosynthetic genes is still not routine, so there remains much more to be learned about biosynthetic pathways as well as about BGC regulation in actinomycetes. However, the field now has a well-established set of genetic tools that greatly facilitate future work. Accurate analytical methods are of great importance, as comparative metabolic profiling is necessary for detection of differences between wild-type strains and their mutants.

## 5. Metabolomics for the Discovery of New Antibiotics Produced by Actinomycetes

In the pre-genomic era of antibiotic discovery most of the identified molecules were isolated by a classical “top-down” approach, which implies that they were produced in sufficient amount for their subsequent detection [219]. Since this conventional approach more and more failed because of frequent rediscovery of known metabolites, new approaches were required to systematically access novel antibiotics [220]. The increasing availability of genomic data for producers of natural products led to the recognition of the hidden potential of microbes to produce an even bigger variety of secondary metabolites as originally expected, and this notion changed the overall strategy for antibiotic discovery [211,221,222,223,224]. Genome mining and metagenomic analysis were added to the portfolio of methods for the prioritisation of newly isolated microbial strains, as well as for realising the previously unseen biosynthetic potential of already analysed strains [85,225]. As powerful as these *in silico* strategies are, they can only succeed when combined with analytical chemistry techniques, particularly high-resolution mass spectrometry and NMR (nuclear magnetic resonance spectroscopy) for structure elucidation of new molecules [226]. To access the full biosynthetic potential of microbes—in particular actinomycetes—as antibiotic producers, metabolomics is one of the most important tools due to its capability to systematically assess all primary and secondary metabolites in a biological sample [227,228]. The use of hyphenated techniques and the extension of the scope of already existing technologies in combination with advanced data evaluation set the stage to enlarge the window of observable metabolites. In this part of the review, we therefore highlight advances in the field of analytical chemistry including imaging mass spectrometry (IMS), liquid chromatography coupled to nuclear magnetic resonance spectroscopy (LC-NMR) and supercritical fluid chromatography (SFC). Molecules detected with IMS in the past 10 years are summarised and differences in ionisation methods compared. LC-NMR and SFC are briefly outlined, as they represent to date underexploited technologies in the field of actinomycetes compound discovery, showing already promising results in other natural product applications. Moreover, an overview is given about conceptual improvements for natural products dereplication using metabolomic data during the last 10 years.

### 5.1. Innovations in Analytical Instrumentation for Natural Product Discovery

#### 5.1.1. Imaging Mass Spectrometry (IMS)

**Figure 10:**
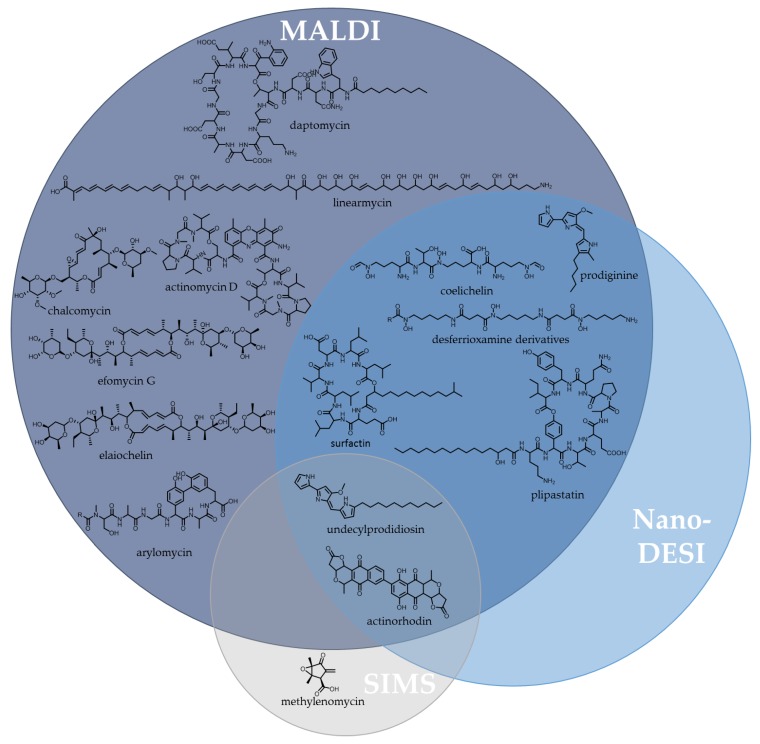
Natural products produced by actinomycetes detected and annotated by IMS. Fifteen known bioactive compounds were identified in different co-cultivation experiments. Only methylenomycin [239] exclusively had been detected using SIMS, whereas all molecules identified with Nano-DESI were also detectable by MALDI-TOF. Undecylprodigiosin [239,240] and actinorhodin [239,240] could be identified by all of the three methods. Prodiginine [240], coelichelin [240], different desferrioxamine derivatives [240], surfactin and plipastatin [241,242] could be assigned by NanoDESI and MALDI-TOF. Chalcomycin [243], daptomycin [243], actinomycin D [244], efomycin G [244], elaiochelin [244], linearmycin [243,245] and arylomycin [231] were only detected by MALDI-TOF.

The introduction of ultra-high performance liquid chromatography (UHPLC) in combination with high-resolution MS detectors such as time of flight mass spectrometry (TOF-MS) remains one of the breakthroughs for natural product discovery [229]. This combination has since provided a rapid and robust analysis of the chemical composition of microbial extracts [16,230]. One of the main limitations of this approach comprises the use of a mixture of cells in different growth states for preparation of extracts for LC-MS measurement, wherefore chemotypes cannot be connected to phenotypes [231]. Among the Gram-positive bacteria, actinobacteria show the greatest morphological differentiation. They are able to form complex structures such as spore chain, sporangia or substrate mycelia with long-branching hyphae [232]. The impact of specific phenotypes on the antibiotics production was strikingly shown by Onaka et al. [233] for *Streptomyces endus* S-522: mycolic acid-containing bacteria such as *Tsukamurella pulmonis* stimulate the antibiotic production of other actinomycetes when grown together on agar-plates. Following this co-cultivation approach, Onaka et al. [233] were able to isolate alchivemycin A, an antibiotic unknown before. Modern mass spectrometry ionisation techniques such as nanospray desorption electrospray ionisation (NanoDESI), matrix-assisted laser desorption electrospray ionisation (MALDI-TOF) and secondary ion mass spectrometry (SIMS) imaging opened a way to further investigate the precise mechanism of these cell-to-cell contact interactions [234,235]. Even though IMS is nowadays mostly used for discovery of disease-related biomarkers, it has also proven useful for the detection of natural products. Figure 10 gives an overview of anti-infectives produced by actinomycetes detected by imaging mass spectrometry in the past 10 years.

Interestingly MALDI was the ionisation method of choice in most of the experiments, even though utilisation of a matrix for covering the sample and to adsorb laser energy comes with more time-consuming sample preparation [235]. A reason for this might be that MALDI is currently the most widely accessible IMS technique and provides soft ionisation. This, in turn enables high-resolution mapping of ions within a sample without destroying sensitive molecules [235,236]. NanoDESI belongs to the so-called soft ionisation methods as well, but is a rather new technique compared to MALDI. Desorption electrospray ionisation (DESI) is based on a two capillary system building a small bridge of charged solvent on the sample surface that absorbs the analytes and directs them into the atmospheric inlet of a mass spectrometer [236]. Compared to the quite robust MALDI, DESI is accompanied by great technical challenges since obtaining an ideal analyte signal relies on a big variety of geometrical and instrumental parameters [237]. Even though SIMS is the oldest IMS techniques out of the three methods compared here, it only plays a subordinate role for natural product discovery. This is very likely caused by the fact that a high degree of optimisation is needed to record useful data. Furthermore, measurements of intact natural products is often not possible because of the requirement for a direct ion beam that is focused on the sample surface [235].

How IMS can be successfully implemented for natural product discovery was shown by Kersten et al. [238] through their application of a “peptidogenomic” workflow. MALDI-TOF MS and subsequent MS^n^ sequence tagging was used to match the mass spectrometry data in an iterative approach to the genome-derived peptide structures generated with NRP prediction tools. Limiting the *m*/*z* range to 1500–5000 Da, they were able to detect nine novel lassopeptides from seven genome-sequenced *Streptomyces* strains, as well as the already known stendomycin produced by *S. hygroscopicus* ATCC 53653 [238].

IMS has proven its capability to detect various classes of known natural products produced by actinomycetes. It is an important tool for the direct observation of interspecies interactions and can be used for the detection of novel antibiotics. Nevertheless, most publications reporting the use of IMS focused on known metabolites and even though unassigned masses have been detected, the corresponding compounds still await their isolation and structure elucidation.

#### 5.1.2. Liquid Chromatography Coupled to Nuclear Magnetic Resonance Spectroscopy (LC-NMR)

MS and NMR are sharing the first place of most important technologies for identification and structure elucidation of new natural products [246]. Whilst NMR is commonly used for pre-purified samples, its hyphenation with liquid chromatography expands its application towards the screening stage in natural products discovery. Compared to the combination of liquid chromatography with mass spectrometry (LC-MS), NMR is limited by lower sensitivity but offers the possibility to provide important structural information on-line that are not accessible by MS. It was already proposed for many years, that a combination of NMR and LC (hence LC-NMR) could make structural information available earlier in the discovery workflow compared to classical procedures utilising various steps of fractionation, purification and subsequent structure elucidation using different spectroscopic methods (whereas NMR is usually the final step) [247]. Nevertheless, only the implementation of flow-through probes such as SPE-NMR or CapNMR and technical advantages in NMR spectrometer field strength in the last decades finally could pave the way into the natural products labs for LC-NMR technology [248]. LC-NMR has already proven its value for dereplication of plant extracts for various compound classes such as alkaloids [249,250], phenolic compounds [251,252], isoflavones [253], flavonoids [254] and lignans [255]. Johansen et al. [256] described in 2011 the characterisation of 15 compounds, of which four have not been previously identified, in the extract of the safflower *Carthamus oxyacantha*. They used a combined HPLC-PDA-HRMS-SPE-NMR approach allowing the preselection of peaks observed by PDA-HRMS on a SPE cartridge for subsequent NMR analysis. A following microfractionation in 96-well plates for bioactivity assays as described by Lang et al. [257] would complete a full automated screening process with maximum information about the composition of extracts. Consequently, Lang et al. classified LC-NMR as an evolving trend in the dereplication of fungal extracts.

For bacterial extracts, identification and structure elucidation of six linear peptides (macyranones A–F) from a myxobacterial extract was performed by Keller et al [258] using LC-SPE-NMR-MS techniques in 2015. Furthermore, Lin et al. [259] successfully used a LC-MS-NMR platform for the identification of four known compounds from a single LC injection of a cyanobacterial extract and additionally identified one new bioactive compound in the extract. With regards to actinobacteria LC-NMR has apparently been used only for structure elucidation of four new polyketides (granaticin C and metenaticin A–C) by Pham et al. [260] from a *Streptomyces violaceoruber* extract back in 2005. The finding that LC-NMR did not seem to be used frequently for the isolation and direct structure elucidation of natural products from microbial extracts in the past 10 years may be explained at least in part by the fact that most compounds produced in sufficient amount for identification by NMR—notably being much less sensitive than MS—are already known and for that matter not a target for structure elucidation. Nevertheless, for the investigation of newly isolated strains from new genera, species and families that are very likely to produce chemical diverse unknown metabolites, LC-NMR should be considered a feasible screening strategy [15].

#### 5.1.3. Super Critical Fluid Chromatography (SFC)

Liquid and gas chromatography are irreplaceable instruments for the separation of highly complex bacterial crude extracts [261,262,263]. Another evolving analytical separation technique that has already proven its impact in phytochemistry is supercritical fluid chromatography (SFC) [264]. SFC uses supercritical fluids, most commonly CO_2_, as mobile phase. In this stage, the fluids uniquely combine two of the most desirable features of a mobile phase: high dissolving capabilities but densities comparable to a liquid. As orthogonal method to liquid chromatography it offers the possibility to cope with metabolites not separable by normal phase or reversed phase liquid chromatography [264,265,266,267]. The use of co-solvents such as alcohols extends the spectrum of compound mixtures accessible by SFC. Almost all stationary phases used in HPLC are also feasible for SFC and diverse detectors such as diode array detector (DAD), evaporative light scattering detector (ELSD), MS, DC or charged aerosol detector (CAD) can be fitted to the instrument [268,269]. For plant extracts SFC has already been used for the isolation of nonpolar compounds such as terpenes [270] or flavonoids [271] but it also has proven to be a useful tool for the isolation of more polar natural products such as carsonic acid from rosemary extracts [272] and valerenic acid from *Valeriana officinalis* [273]. Notwithstanding, to our knowledge SFC has never been used for the discovery of new antibiotics from actinomycetes. In consideration of the wide field of natural products it has been used, it certainly will not take long until first research groups will successfully employ SFC for the separation of microbial natural products.

### 5.2. Dereplication Using Metabolomic Data

Metabolomics is the systematic acquisition of small molecules produced by a biological system at a certain point in time. Increasing resolution of the data acquired by state-of-the-art technologies produces an expanding volume of data hereby [274]. To evaluate these data in the best way, dereplication using bioinformatics tools is indispensable [275]. First introduced by Beutler et al. [276] back in 1990 as “a process of quickly identifying known chemotypes” used for the identification of compounds from bacterial extracts responsible for a biological activity in a simple phorboldibutyrate (PDBu) receptor binding assay, the term “dereplication” today covers various strategies and different fields of research from pharmacology, chemistry, plant sciences to biotechnology and food science [226]. One of the main issues in the field of natural product antibiotic discovery is the high rediscovery rate, which can only be limited by careful dereplication. Chanana et al. [277] made the statement, that it is more likely to identify a known metabolite from actinobacteria than finding a new one which is certainly true as many academic and industrial groups have worked on actinomycetes for decades. This group used comprehensive principal component analysis (PCA) for the prioritisation of most interesting bacterial strains and molecules and were therefore able to isolate two novel natural products from an *Actinomadura* sp. (strain WMMB-449) and patented another one [277]. For the prioritisation of bacterial extracts showing promising antibacterial activities it is crucial to sort out extracts containing known metabolites possibly responsible for the observed activity [278]. In-house databases—containing as many chromatographic data from known analytes as possible—provide the advantage of being perfectly matched to the conditions used for the sample of interest, but are difficult to keep up-to-date due to the requirement of continuous manual curation. Commercially available libraries such as Dictionary of Natural Products [279], AntiBase [280], MarinLit [281] and MS databases such as MassBank [282], Metlin [283], mzCloud [284] and ReSpect [285] certainly are useful sources, but are either not freely available, do not contain MS data or do not allow customised use of its reference library. In addition, connectivity between these databases is rather poor, leading to an unpredictable degree of overlap. Dereplication by database search is therefore a cumbersome and time-consuming procedure and last but not least an expensive one [286]. A better connection between already existing libraries, lowering the access barrier and a more intense data exchange between groups working in the field of natural products is strongly required. An important first step in the direction of better data exchange is Global Natural Products Social molecular networking (GNPS), an open-access knowledge platform for sharing tandem mass spectrometry data [287]. In the course of only a few years, GNPS has already proven its high impact for the natural product community. Crüsemann et al. [288] for example used GNPS for the analysis of 146 marine *Salinispora* and *Streptomyces* strains. They were able to identify 15 molecular families of diverse natural products and showed that GNPS is in principle ready for increased throughput screening thereby [288]. However, the simple comparison of MS/MS spectra with spectra deposited in the GNPS is not yet sufficient for a comprehensive analysis of secondary metabolome data. Algorithms such as DEREPLICATOR [289] or the just recently published VarQuest [290] were designed to expand the library search to identify variants of known peptidic natural products. The complete workflow from genome sequence to the putative biosynthetic gene cluster and its product with the associated spectra would ideally be done in a one-step procedure for high-throughput data analysis. Several tools have taken on this challenge and specifically address the connection of genes with secondary metabolites. Table 1 is a comparison of six different bioinformatic software packages comprising a structure prediction tool for chemical products derived from biosynthetic gene clusters: PRISM 3, SeMPI, antiSMASH 4.0, Pep2Path, RiPPquest and NRPquest. As some of them are designed for much broader analysis of genomic data, we would like to point out that they are henceforth just compared concerning structure prediction. Tools with more than one software release were summarised with functions available in the newest version to date.

AntiSMASH 4.0 presumably is one of the prediction tools that offers the broadest range of *in silico* analysis. Biosynthetic gene clusters are predicted using hidden Markov models and manually curated BLAST databases for identification of biosynthetic domains involved in secondary metabolite production. Subsequently the domains are arranged into clusters and chemical structures of the products are predicted. The hits are finally compared with a library of known natural products [122,295]. PRISM 3 follows a similar concept. However recent software releases of AntiSMASH were more focused on improving the genomic data evaluation and accessibility of prediction for additional cluster types such as terpenes [121,122,292]. Therefore, PRISM 3 remains the most promising bioinformatics tool for prediction of tailoring reactions on NRPS and PKS products [184,185]. Other bioinformatic tools such as Pep2Path, RiPPQuest and NRPQuest are limited to peptidic natural products [183,293,294]. Peptidic natural products (PNPs) however feature characteristic MS/MS-fragmentation patterns and usually reliable ionisation behaviour [293].

MS/MS metabolomic data can be used to complement the theoretical *in silico* prediction of chemical products produced by biosynthetic gene clusters. Thus Liu et al. [296] were able to identify the stenothricin biosynthetic gene cluster in *Streptomyces roseosporus* in 2014. Peptidogenomics in this manner is a powerful tool for full-automated high-throughput screening for bacterial extracts using both experimental and *in silico* data. Another elegant way to correlate natural products to their corresponding BGCs is using the tools mentioned above to predict specific physicochemical properties. The macrolactam salinilactam isolated from *Salinospora tropica* could be identified in a crude extract by searching for characteristic UV absorption of polyenes [297]. Since detection by UV absorption is not very specific when applied alone, rediscovery rate with such an approach is high. Therefore, MS-based methods using the identification of chlorine, bromine, fluorine and sulphur in a molecule by their characteristic isotopic pattern in combination with the exact molecular mass are more specific for the identification of secondary metabolites. In particular, this method has proven to be useful for the identification of related compounds as exemplified by the isolation of the thioholgamides from a crude extract of *Streptomyces malaysiense* MUSC 136 57. A combination of search for BGCs related to the one of thioviridamide and prioritising of potential products by their characteristic isotopic pattern revealed these natural products with promising cytotoxic activity [298]. Additionally, feeding experiments of isotopically labelled precursors are useful to narrow down potential products of the BGC present in the crude extract and provide further information about the biosynthesis of the secondary metabolite.

### 5.3. Conclusions

The widespread availability of high-resolution mass spectrometry in the past two decades has been a milestone for analytical chemistry not only for actinobacterial extracts. Especially in combination with HPLC this technology provides the opportunity for rapid and robust analysis of the bacterial metabolome. This advance increased the quality as well as the quantity of data acquired on a daily basis. The focus of metabolomics-driven microbial natural product research therefore was on extending the scope of molecules accessible to high resolution MS and subsequent data analysis including dereplication. IMS, LC-NMR and SFC are recent technical improvements useful for the characterisation of bacterial extracts. Although most of the modern analytical systems are commercially available, the application of most of the presented techniques was focused on one specific research field such as phytochemistry for SFC. Mass spectrometry and NMR still form the stable foundation for metabolomics of microorganisms. Their sphere of influence has already been extended by hyphenated techniques such as LC-NMR and will be certainly further increased by the combination with underrepresented techniques such as SFC in the coming years. The rising quality and quantity of metabolomic data obtained with hyphenated techniques in the past decades increased the importance of bioinformatics tools to organise and prioritise the data. There are various tools published, that either can be used for library matching or even for structure formula prediction. One big issue in natural product research is data exchange within the different research groups to hinder rediscovery of already known compounds. The establishment of the GNPS that allows sharing MS^2^-data of natural products and other raw data is a good start towards a better exchange within the community. Nevertheless, as much as technical improvements can only unfold their full potential when combined with bioinformatic tools, they are more powerful, when genetic information is included in the search for new antibiotics from actinobacteria.

## 6. Summary and Conclusions

**Figure 11:**
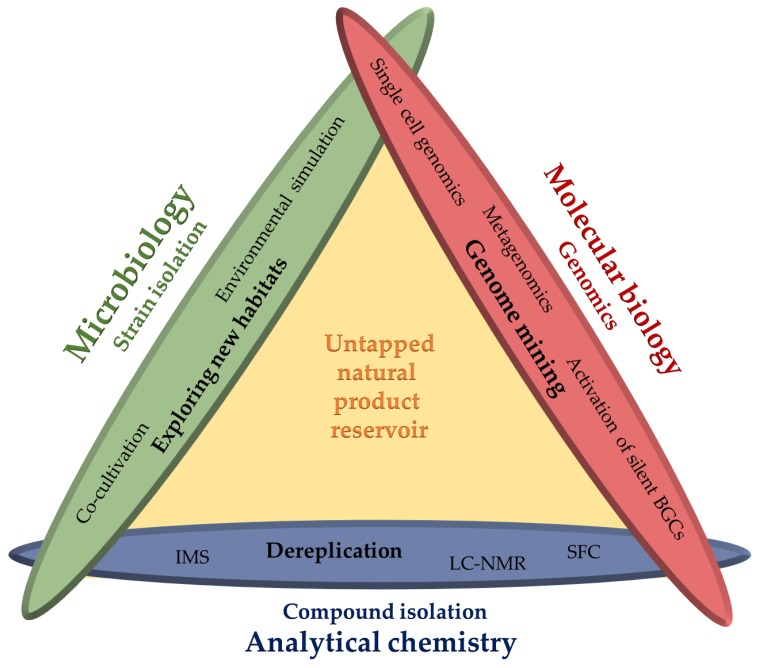
Interplay of disciplines for the exploitation of the untapped natural product reservoir with major developments in the past 10 years covered by this review.

Actinomycetes are still a prolific source for natural products with intriguing bioactivities and inspiring chemical characteristics as exemplified by the new antimicrobial agents discovered in the past 10 years [164]. Considering that the majority of microbial secondary metabolite producers are not culturable under laboratory conditions yet, the potential for discovery of novel actinomycete metabolites is promising and far from exhausted [14]. However, as actinomycete research has been extensively performed in the past by academic and industrial groups, chances for the identification of novel chemistry may be higher in currently less studied microbial resources such as cyanobacteria or myxobacteria [299,300]. Natural product research is fundamentally dependent on the interplay of different disciplines of natural sciences. Particularly, microbiology for the isolation of new secondary metabolite producers, molecular biology for the characterisation of the biosynthetic machinery and analytical chemistry for identification and isolation of natural products form the basis for the characterisation of novel compounds from actinobacteria (Figure 11).

In the past years, focus on natural product discovery from actinomycetes shifted from the extensively investigated soil-dwelling isolates towards underexplored habitats of rare actinomycetes from unusual ecosystems. This strategy has been shown to give an impact on the discovery platform for novel compounds with promising bioactivities despite the fact that a large amount of the reservoir of habitats still awaits exploration [301]. The full potential of the natural product “treasure trove” of environmental soil samples from exotic or conventional habitats is still underexplored, since present isolation procedures fail to access the uncultured microbial majority. Nevertheless, the “genomic revolution” has revealed that actinobacteria carry more biosynthetic gene clusters in their genome than secondary metabolites found under standard laboratory conditions. This finding shows that harvesting novel strains just for few compounds understates their immense potential. The simulation of environmental conditions to mimic essential aspects of the environment extended the scope of microorganisms culturable under laboratory conditions [35]. Although technologies such as the iChip platform have shown great potential for increasing throughput and improving the isolation of previously uncultured bacteria, novel microbiology-based methods are still underrepresented in the natural product discovery pipeline. Genomics as a field that has seen tremendous development over the past 10 years, in contrast has rapidly triggered the establishment of several innovative methods to explore the diversity and distributions of BGCs encoding natural products on the genetic level [302]. To overcome obstacles of conventional isolation procedures and access the uncultured microbial majority of rare actinomycetes, metagenomic approaches have been developed. With the growing underlying in-depth knowledge of biosynthetic gene organisation, affordable whole genome sequencing technologies and prediction methods of the resulting natural product structure, “silent“ BGCs can be leveraged to yield novel compounds. BGCs from genetically non-manipulable or uncultured actinomycetes can be accessed via heterologous expression through capturing the BGC and refactoring the gene arrangement. The combination of methods from synthetic biology, biotechnology and genetics is thus sets to expand Nature’s chemical diversity through production of “unnatural” natural products and naturally expression of downregulated BGCs [303,304]. Despite the enormous impact of genomics, we must urgently remind ourselves that all sequence-based developments need to be supported by microbiology and analytical chemistry for production and isolation of compounds. In the field of analytical chemistry, advances have been made for the development of bioinformatic tools for an easier and faster dereplication. Furthermore, applied analytical chemistry in the field of natural product research is up to date focused on retro-biosynthetic logic to correlate secondary metabolites to their BGCs. Technologically, hyphenated techniques such as LC-NMR and separation techniques such as SFC have entered the stage more recently for the discovery of novel antibiotics but are not fully utilised for the actinobacterial natural product research yet [247]. Furthermore, imaging mass spectrometry has proven its ability to directly provide deep insights in the connection between phenotypic traits and secondary metabolism, but upscaling remains a formidable challenge [242]. On this occasion, advances in microbiological cultivation strategies are required to utilise the full potential of this method for the discovery of antibiotics. This is just one of many examples where improvements in one of the three key disciplines in natural product research will have synergistic effects when efficiently combined with other methods. Only the combination of technologies and exchange of knowledge between microbiology, molecular biology and analytical chemistry will help to not only scratch the surface of the untapped natural product reservoir but comprehensively exploit its full potential.

## Figures and Tables

**Table 1:** Comparison of six different bioinformatic tools for structure prediction of chemical products derived from biosynthetic gene clusters: PRISM 3, SeMPI, antiSMASH 4.0, Pep2Path, RiPPquest and NRPquest. Tools with more than one software release were summarised with functions available in the newest version to date.

Acronym	Input	Output	Type of BGC	Highlights & Limitations	Accessibility	Source
**PRISM 3**	Genome sequence	Gene clusters	NRPS, PKS	Tailoring reactions implemented ^1^	Open-source web application	[184,185]
**SeMPI**	Genome sequence (*also raw DNA code*)	Domains and 10 best matching compounds	Type I PKS	Generation of the non-modified PKS products ^1^	Open-source web application	[291]
**antiSMASH 4.0**	Genome sequence	NRPS/PKS domains, chemical structure prediction, Cluster Blast	NRPS, PKS, RiPPs terpenes	Terpene prediction Trans AT PKS domain alignments ^1^	Open-source webserver	[121,122,292]
**Pep2Path**	Genome sequence mass shifts or amino acid-sequence	Tag-BGC alignment and scoring	NRPS, RiPPs	Automatic identification of BGC corresponding to mass shift sequence or amino acid-sequence tags ^2^	Open-source application	[293]
**RiPPquest**	Genome sequence MS/MS dataset	Peptide-spectrum match (*p*-value)	RiPPs	Molecular network analysis using input from various MS/MS datasets possible ^3^	Open-source application, (implemented in GNPS)	[294]
**NRPquest**	Genome sequence MS/MS dataset	Spectral network of matching peptides with *p*-values	NRPs	Molecular network analysis of various MS/MS datasets possible ^4^	Open-source application	[183]

^1^ No matching with experimental MS/MS data; ^2^ Limited to peptide natural products; ^3^ Limited to RiPPs; ^4^ Limited to NRPs.
